# PD-1 or CTLA-4 blockade promotes CD86-driven Treg responses upon radiotherapy of lymphocyte-depleted cancer in mice

**DOI:** 10.1172/JCI171154

**Published:** 2024-02-13

**Authors:** Elselien Frijlink, Douwe M.T. Bosma, Julia Busselaar, Thomas W. Battaglia, Mo D. Staal, Inge Verbrugge, Jannie Borst

**Affiliations:** 1 Division of Tumor Biology and Immunology and Oncode Institute, The Netherlands Cancer Institute, Amsterdam, Netherlands.; 2 Department of Immunology and Oncode Institute, Leiden University Medical Center, Leiden, Netherlands.; 3 Division of Molecular Oncology and Immunology and Oncode Institute, The Netherlands Cancer Institute, Amsterdam, Netherlands.

**Keywords:** Immunology, Oncology, Cancer immunotherapy, Costimulation, Radiation therapy

## Abstract

Radiotherapy (RT) is considered immunogenic, but clinical data demonstrating RT-induced T cell priming are scarce. Here, we show in a mouse tumor model representative of human lymphocyte–depleted cancer that RT enhanced spontaneous priming of thymus-derived (FOXP3^+^Helios^+^) Tregs by the tumor. These Tregs acquired an effector phenotype, populated the tumor, and impeded tumor control by a simultaneous, RT-induced CD8^+^ cytotoxic T cell (CTL) response. Combination of RT with CTLA-4 or PD-1 blockade, which enables CD28 costimulation, further increased this Treg response and failed to improve tumor control. We discovered that upon RT, the CD28 ligands CD86 and CD80 differentially affected the Treg response. CD86, but not CD80, blockade prevented the effector Treg response, enriched the tumor-draining lymph node migratory conventional DCs that were positive for PD-L1 and CD80 (PD-L1^+^CD80^+^), and promoted CTL priming. Blockade of CD86 alone or in combination with PD-1 enhanced intratumoral CTL accumulation, and the combination significantly increased RT-induced tumor regression and OS. We advise that combining RT with PD-1 and/or CTLA-4 blockade may be counterproductive in lymphocyte-depleted cancers, since these interventions drive Treg responses in this context. However, combining RT with CD86 blockade may promote the control of such tumors by enabling a CTL response.

## Abstract 2

In lymphocyte-depleted cancer, PD-1 and CTLA-4 blockade promote radiotherapy-induced Treg responses in a CD86-dependent manner and prevent CD8+ T-cell mediated tumor control.

## Graphical abstract

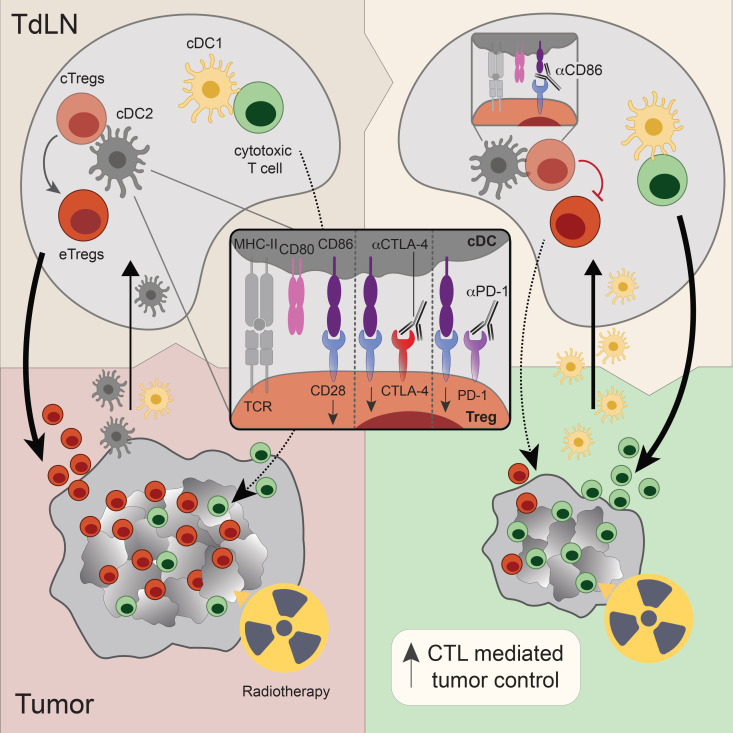

## Introduction

Immunotherapy by antibody-based immune checkpoint blockade (ICB) is a new treatment modality for multiple cancer types. However, only a minority of patients experience durable clinical responses (1), partly due to diverse tumor immune infiltrates (2). Recent pan-cancer transcriptome analyses have elucidated the immune cell compositions of most human solid cancer types, defining intratumoral immune cell types and states associated with a good or bad prognosis (3, 4). These analyses have categorized clinically defined cancer types into subsets with different immune infiltrates (3–5). Studies in mouse models have clarified that such infiltrates develop in dialogue between the tumor, its draining lymph nodes (dLNs), and circulating immune cells (6). Tumors generally exhibit either infiltration of T cells associated with good prognosis, or an absence of such T cells. In the latter case, tumors are often rich in myeloid cells and contain a bad-prognosis T cell infiltrate dominated by Tregs (3–5). ICB responsiveness is typically linked to tumor types infiltrated by good-prognosis, effector-type CD4^+^ and CD8^+^ T cells (4). The presence of good-prognosis T cells in tumors depends on the presence of tumor antigens but also on favorable communication between the tumor and its dLN via conventional dendritic cells (cDCs) (7). This communication is primarily shaped by tumor genetics, including oncogenic driver pathways (8).

Interventions should elicit de novo T cell responses to achieve clinical benefit in cancers devoid of effector T cells (9). In attempts to accomplish this, ICB has been combined with radiotherapy (RT) in multiple clinical trials (10). This combination is attractive for several reasons: (a) RT-induced tumor cell death reduces tumor burden, potentially relieving systemic immune suppression; (b) RT can modulate the tumor microenvironment (TME), making it more permissive for T cell–mediated tumor destruction (11); and (c) RT can support systemic antitumor immunity by generating new tumor-specific T cell responses in the tumor-dLNs (TdLNs), a process called T cell priming. It is proposed that RT can prime systemic antitumor T cell responses, on the basis of theory and observations in mouse models (12–14). Upon RT-induced tumor cell destruction, cell debris containing tumor-derived antigens and danger-associated molecular patterns (DAMPs) occurs (15). Locally, migratory cDCs engulf this debris, migrate to TdLNs, and initiate T cell responses. To prime CD8^+^ T cells, the cDC1 subset is required that excels at cross-presenting peptides from phagocytosed proteins in MHC class I (MHC-I) molecules. Activated cDC1s also provide specific costimulatory and cytokine signals, instructing CD8^+^ T cells to expand and differentiate into competent cytotoxic T lymphocytes (CTLs) (16). The potential of RT to induce a systemic T cell response predicts that it may potentiate abscopal effects, i.e., tumor regression outside the field of radiation. Clinically, such observations are extremely rare (17), indicating impediments in this process (11). In certain immunogenic mouse tumor models, RT can induce T cell infiltration of the irradiated tumor, as well as an “abscopal” tumor implanted on a nonirradiated site in the same mouse (18–20).

However, clinical effects of combining RT with CTLA-4 or PD-1 targeting ICB are disappointing (10, 21–24). For example, RT as induction treatment did not enhance PD-1 blockade efficacy in patients with metastatic triple-negative breast cancer, nor did it improve T cell infiltration into the TME (21). We propose that the immune cell composition of the tumor, as dictated by its dialogue with the TdLN, is decisive for the success of RT/ICB combinations. Certain mouse tumor models spontaneously become infiltrated with tumor-specific effector T cells and regress upon RT alone (25) or in combination with ICB (26), without requiring de novo T cell priming. In such T cell–infiltrated tumors, RT apparently enables tumor-infiltrated T cells to exert their effector functions locally. However, in lymphocyte-depleted tumor types that lack preexisting tumor-specific effector T cells, RT must induce new T cell priming to enable T cell–mediated tumor control. Lack of antigens, insufficient cDC activating signals (27), and/or tumor-imposed immunosuppression can hamper this process (28).

In this study, we delineate how the T cell response to RT may proceed in lymphocyte-depleted cancers. For this purpose, we defined a mouse tumor model representing human lymphocyte–depleted cancer by bioinformatics analysis and used it for detailed analysis of RT-induced T cell immunity and the effect of ICB. We found that this tumor type spontaneously induced priming and tumor infiltration by effector phenotype, thymus-derived (FOXP3^+^Helios^+^) Tregs, which was exacerbated by RT and prevented CTL-mediated tumor control. Counterintuitively, antibody-mediated blocking of the coinhibitory receptors CTLA-4 or PD-1 further increased this Treg response and antagonized tumor regression.

Recent work has indicated that both CTLA-4 and PD-1 blockade enable CD28 costimulation of T cells. CD28 signals amplify T cell receptor (TCR)/CD3 signals to promote the expansion of newly activated CD4^+^ and CD8^+^ T cells (29). CTLA-4 is constitutively expressed on Tregs and downregulates the CD28 ligands CD80 and CD86 on cDCs (30). Therefore, CTLA-4 attenuates the ability of cDCs to induce CD28 costimulation of conventional, nonregulatory T cells (Tconvs) (30). PD-1 is associated with the SHP2 tyrosine phosphatase that extinguishes CD28 signals in *cis* (31). Thus, CTLA-4 and PD-1 use different mechanisms, but both control T cell responses by suppressing CD28 costimulation. We discovered that in the lymphocyte-depleted cancer model, CD28 costimulation enabled by ICB drove the RT-induced Treg response. Selective blockade of the CD28 ligand CD86 inhibited the Treg response and promoted CTL priming and tumor control. We therefore advise that combining RT with PD-(L)1– and/or CTLA-4–targeting ICB can be counterproductive in lymphocyte-depleted cancers and identify CD86 as an alternative target for ICB in such cases.

## Results

### The RT response is deficient in T cell–depleted human tumor types.

**Figure 1:**
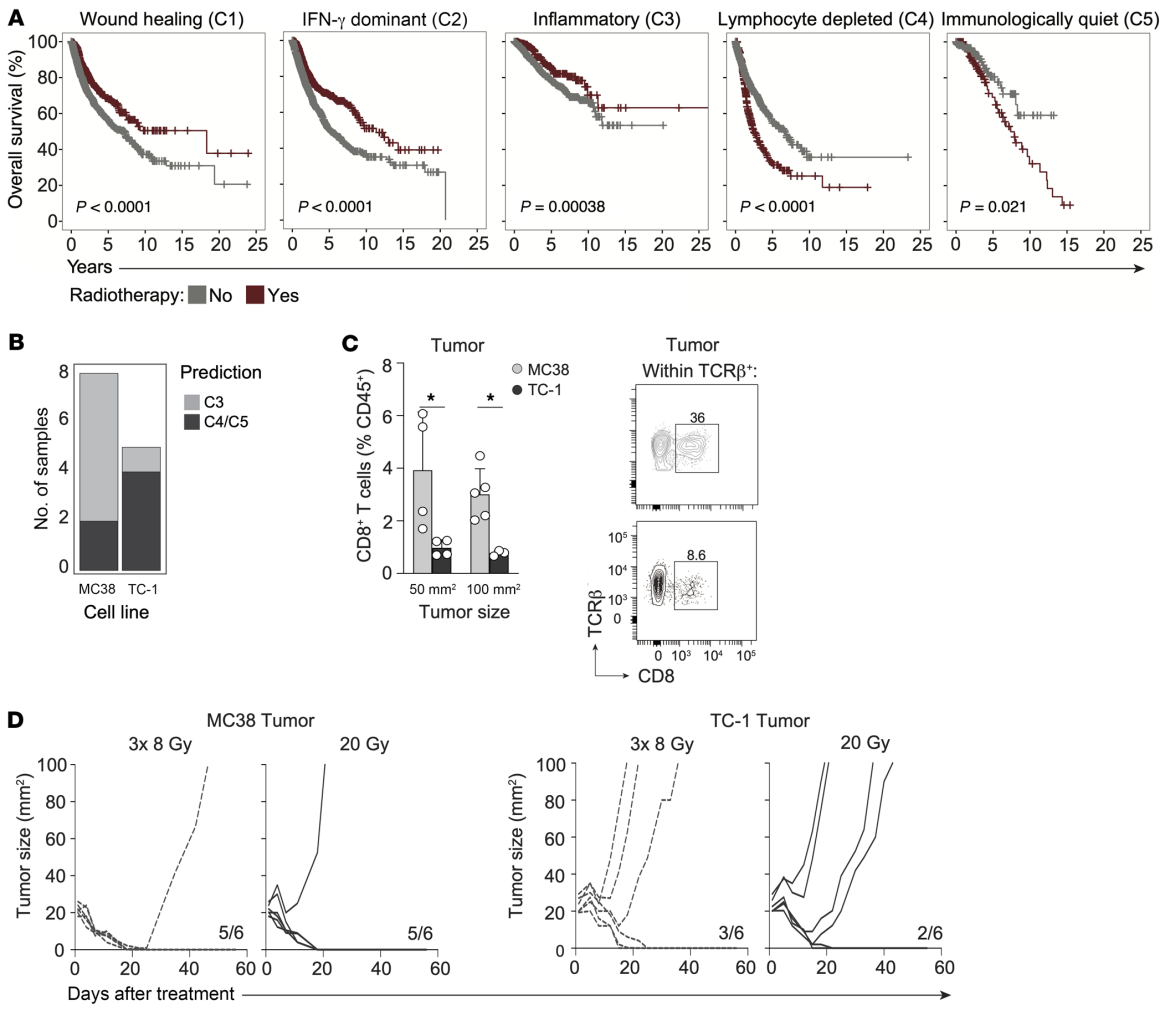
Lymphocyte-depleted (C4/C5) human cancers have suboptimal responses to RT and are modeled by the murine TC-1 tumor. (**A**) Kaplan-Meier OS curves obtained from TCGA for patients receiving RT (red) or not (gray) within the C1 “wound healing” (*n* = 2,136), C2 “IFN-γ dominant” (*n* = 2,296), C3 “inflammatory” (*n* = 1,903), C4 “lymphocyte-depleted” (*n* = 1,055), and C5 “immunologically quiet” (*n* = 354) cancer immune subtypes. *P* values (log-rank) were generated using a Cox proportional hazards model. (**B**) C3 “inflammatory” versus C4/C5 “lymphocyte-depleted” model predictions from transcriptome data on C57BL/6 syngeneic MC38 and TC-1 transplantable tumors. (**C**) Frequency of CD8^+^ T cells among CD45^+^ cells in MC38 (total *n* = 9) and TC-1 (total *n* = 7) tumors measured at the indicated tumor sizes (left) and representative flow cytometric plots (right) depicting the percentage of CD8^+^ T cells within TCRβ^+^ cells in 50 mm^2^ MC38 (gray) and TC-1 (black) tumors. (**D**) Tumor growth curves for mice bearing MC38 (*n* = 6/group, left) or TC-1 (*n* = 6/group, right) tumors that were treated with either 8 Gy over 3 days (3× 8 Gy) or a single dose of 20 Gy RT. Ratios indicate the number of mice among the total number of mice treated that showed full recovery upon RT. Error bars indicate the SD. *P < 0.05, by Mann-Whitney *U* test.

To identify how the tumor immune cell composition influences RT responses in human cancer, we examined the relationship between immune phenotype and RT efficacy in a wide variety of human cancers. Using records from The Cancer Genome Atlas (TCGA), we identified 5 previously characterized pan-cancer immune phenotypes (3) in patients for whom RT status was specified (Supplemental Figure 1, A and B; supplemental material available online with this article; https://doi.org/10.1172/JCI171154DS1). These immune phenotypes are described as “wound healing” (C1), “IFN-γ dominant” (C2), “inflammatory” (C3), “lymphocyte depleted” (C4), and “immunologically quiet” (C5). While RT had a positive effect on overall survival (OS) in tumors classified as C1–3 immune subtypes, RT had a negative effect on OS in the C4 and C5 subtypes (Figure 1A) that are identified by low lymphocyte and high myeloid cell content (3). The remarkably defective response to RT of tumors with a C4 or C5 immune phenotype prompted us to examine the underlying mechanism.

We set out to find a mouse tumor model with a C4/C5-like lymphocyte-depleted phenotype. We trained a K-nearest neighbor (KNN) classifier to distinguish between the C3 versus C4/C5 immune subtypes (Supplemental Figure 1C) and subsequently applied our model to microarray data on murine C57BL/6-derived MC38 and TC-1 tumor models (32). We found similarity between the colon carcinoma cell line MC38 and the C3 subtype and between the lung carcinoma cell line TC-1 and the C4/C5 subtype (Figure 1B). Although both tumors express non-self antigens (33, 34), the MC38 tumor is immunogenic and raises a high T cell infiltrate (20), whereas the TC-1 tumor does not (35). In agreement, MC38 is responsive to ICB (36), whereas TC-1 is not (37). Accordingly, flow cytometric analysis revealed a significantly lower proportion of CD8^+^ T cells in TC-1 tumors compared with MC38 tumors (Figure 1C).

We assessed how MC38 and TC-1 tumors respond to RT using 3 consecutive doses of 8 Gy (3× 8 Gy) or a single dose of 20 Gy, regimens that are immune stimulatory in mouse tumor models (12, 38). Both regimens led to MC38 tumor control but were much less effective in TC-1 tumor control (Figure 1D). This agrees with the finding that the preexisting T cell infiltrate in the MC38 tumor contributes to the RT response (25) and suggests impediments for immune-mediated control of the TC-1 tumor upon RT. We therefore continued our study with the TC-1 tumor to examine the RT-induced T cell response in this representative model of lymphocyte-depleted cancer.

### Despite high myeloid and Treg cell content, the RT response of TC-1 is CD8^+^ T cell dependent.

**Figure 2:**
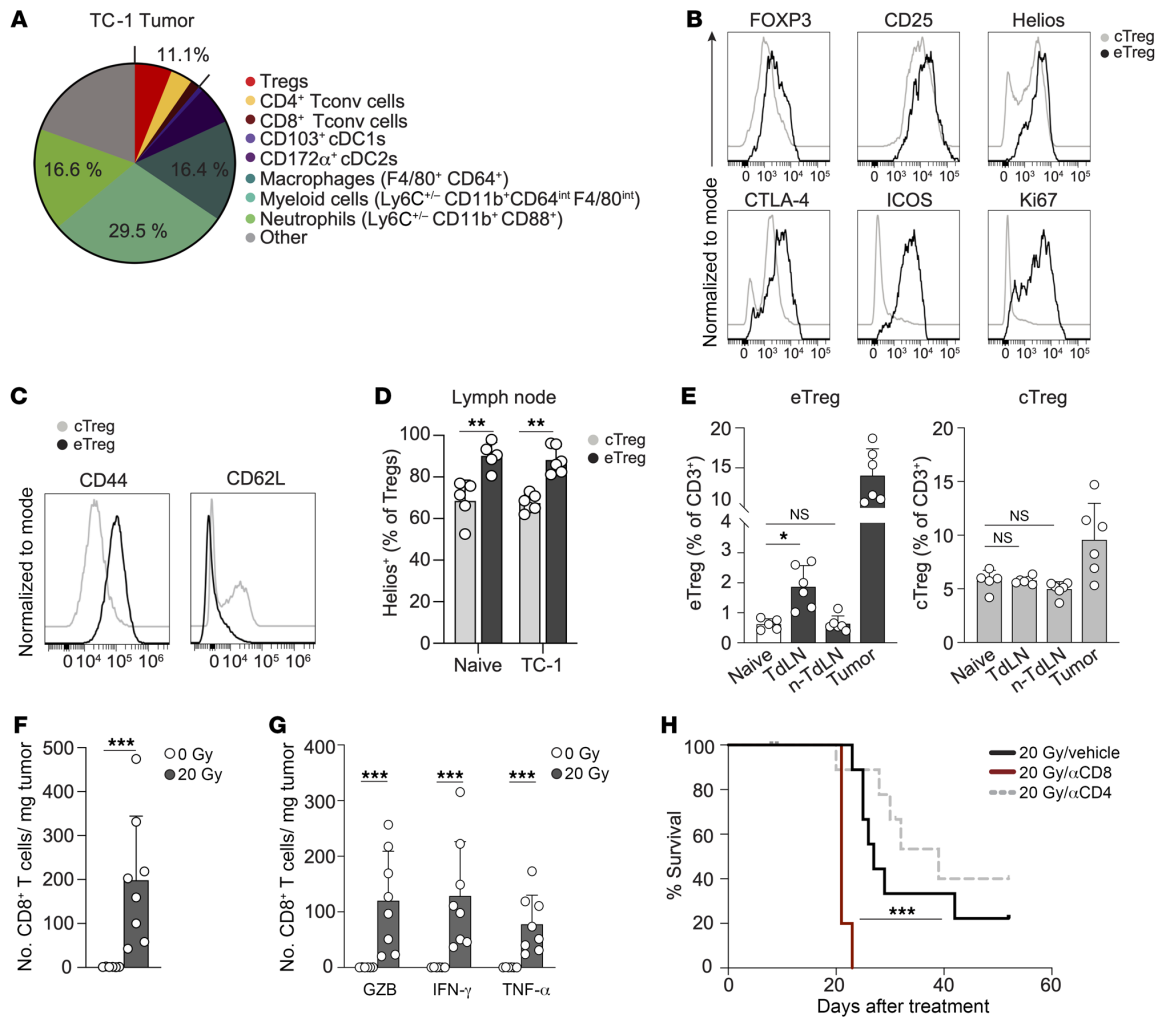
Myeloid cell– and Treg-rich TC-1 tumor shows a CD8^+^ T cell–dependent RT response. (**A**) Frequency of the indicated immune cell populations among CD45^+^ cells measured by flow cytometry in 50 mm^2^ TC-1 tumors (*n* = 6). (**B**–**D**) cTregs and eTregs were defined as indicated in Supplemental Figure 2, B–D, and identified in the TdLN, non-TdLN, and tumor of 100 mm^2^ TC-1 tumor–bearing mice (*n* = 6) and age-matched naive (non-tumor-bearing) mice (*n* = 5). FlowSOM-guided clustering was performed on 5,000 randomly selected cells per sample within the CD3^+^ lymphocyte population. (**B** and **C**) Representative histograms depicting expression of the indicated markers on cTreg and eTreg populations in axillary LNs of naive and TC-1 tumor–bearing mice. (**D**) Frequency of Helios^+^ cells among cTregs and eTregs in axillary LNs of naive and TC-1 tumor–bearing mice. (**E**) Percentage of eTregs (left) and cTregs (right) among CD3^+^ T cells in the indicated tissues. (**F**–**H**) Monitoring by flow cytometry of the CD8^+^ T cell response to 20 Gy RT (*n* = 8) or control (0 Gy, *n* = 6) in TC-1 tumors. n-TdLN, non-TdLN. (**F**) Absolute number of total CD8^+^ T cells and (**G**) GZB-, IFN-γ–, or TNF-α–expressing CD8^+^ T cells per milligram of tumor tissue on after post-RT day 8. IFN-γ and TNF-α levels were measured after in vitro PMA/ionomycin stimulation. (**H**) OS of TC-1 tumor–bearing mice treated with 20 Gy RT on day 0 in combination with vehicle (PBS, *n* = 9) or depleting mAbs specific for CD8 (*n* = 5) or CD4 (*n* = 9). αCD8, anti-CD8 mAb. ***P < 0.001 (Mantel-Cox analysis). Data are from 1 experiment and are representative of at least 2 experiments. Error bars indicate the SD. *P < 0.05, **P < 0.01, and ***P < 0.001, by Kruskal-Wallis test with uncorrected Dunn’s post hoc analysis (**E**) and Mann-Whitney *U* test (**D**, **F**, and **G**).

In the TME of the TC-1 tumor, the T cell compartment, consisting of CD8^+^ and CD4^+^ Tconvs and FOXP3^+^ Tregs, comprised only 11.1% of the CD45^+^ hematopoietic cell infiltrate, as identified by flow cytometry. Conversely, myeloid cells comprised 62.5% of the CD45^+^ cell infiltrate, including macrophages and neutrophils (Figure 2A and Supplemental Figure 2A), consistent with a myeloid-rich, T cell–devoid phenotype (4, 5). The association between Tregs and (suppressive) myeloid cell infiltrates is well described and often linked to (systemic) immunosuppression (6). To characterize the T cell population, we performed detailed spectral flow cytometric analysis of the CD3^+^ lymphocyte population in the tumor, TdLNs, and non-TdLNs. FlowSOM-guided clustering analysis and uniform manifold approximation and projection (UMAP) dimension reduction (Supplemental Figure 2, B and C) identified 7 main clusters, including CD8^+^ and CD4^+^ (FOXP3^–^) Tconvs, proliferating (Ki67^+^) CD8^+^ and CD4^+^ T cells, central (c)Tregs, effector (e)Tregs, and CD4^–^/CD8^–^ T cells. The Tregs that prevent autoreactive Tconv responses at steady state originate in the thymus and reside in secondary lymphoid organs as cTregs. In response to antigen and inflammatory signals, cTregs can expand and differentiate into eTregs that populate peripheral tissues to dampen inflammation (39). The eTreg population was proliferating and had high expression of the effector marker ICOS, alongside the steady-state Treg markers FOXP3, CTLA-4, and CD25. Coexpression of the transcription factor Helios indicated that these eTregs were thymus derived and not peripherally induced Tregs resulting from the conversion of Tconvs into Tregs (40). eTregs displayed high expression of CD44 and lacked CD62L (Figure 2C and Supplemental Figure 2D), confirming their effector phenotype (39). Unlike the eTregs, cTregs, as defined in the TdLN, expressed CD62L, did not proliferate, had no ICOS expression, and had lower expression of the Treg markers (Figure 2, B and C and Supplemental Figure 2D). Helios was expressed in over 70% of cTregs, and this was further enriched in eTregs in both naive and TdLNs (Figure 2D and Supplemental Figure 2D). Quantification of the identified cell populations revealed no increase in proliferating CD8^+^ or CD4^+^ Tconvs in LNs upon TC-1 tumor outgrowth (Supplemental Figure 2E). However, compared with naive mice, the frequency of eTregs , but not cTregs, in the TdLN was significantly increased in tumor-bearing mice, and eTregs were also present in the tumor (Figure 2E). Importantly, the Treg population in the tumor had, overall, a CD44^hi^CD62L^–^ effector phenotype (Supplemental Figure 2D), but given the low levels of ICOS and CTLA-4 and the lack of proliferation, they clustered as cTregs (Supplemental Figure 2, B–D). These data suggest that during its outgrowth, the TC-1 tumor stimulated the expansion and differentiation of Tregs in the TdLN, and these cells also populated the tumor, underscoring the communication between the tumor and the TdLN (6, 41).

Importantly, RT with either 20 Gy or 3× 8 Gy significantly augmented the absolute number of CD8^+^ T cells in the TC-1 tumor (Figure 2F and Supplemental Figure 3A). These tumor-infiltrating CD8^+^ T cells were functional CTLs, as evidenced by the expression of granzyme B (GZB) and the effector cytokines IFN-γ and TNF-α (Figure 2G). Both RT regimens also increased the absolute number of (FOXP3^–^) CD4^+^ Tconvs, albeit to a lesser extent than the increase in the absolute number of CD8^+^ T cells (Supplemental Figure 3B). Systemic depletion of CD8^+^ T cells, but not of CD4^+^ T cells, significantly reduced RT-induced mouse survival (Figure 2H and Supplemental Figure 3, C–E), arguing that the RT-induced CTL response made a major contribution to the control of the TC-1 tumor by RT. This finding suggests that there might be a window of opportunity to improve RT-induced, CTL-mediated control of lymphocyte-depleted cancers.

### RT of the TC-1 tumor induces CTL priming, next to a Treg response that limits tumor control.

**Figure 3:**
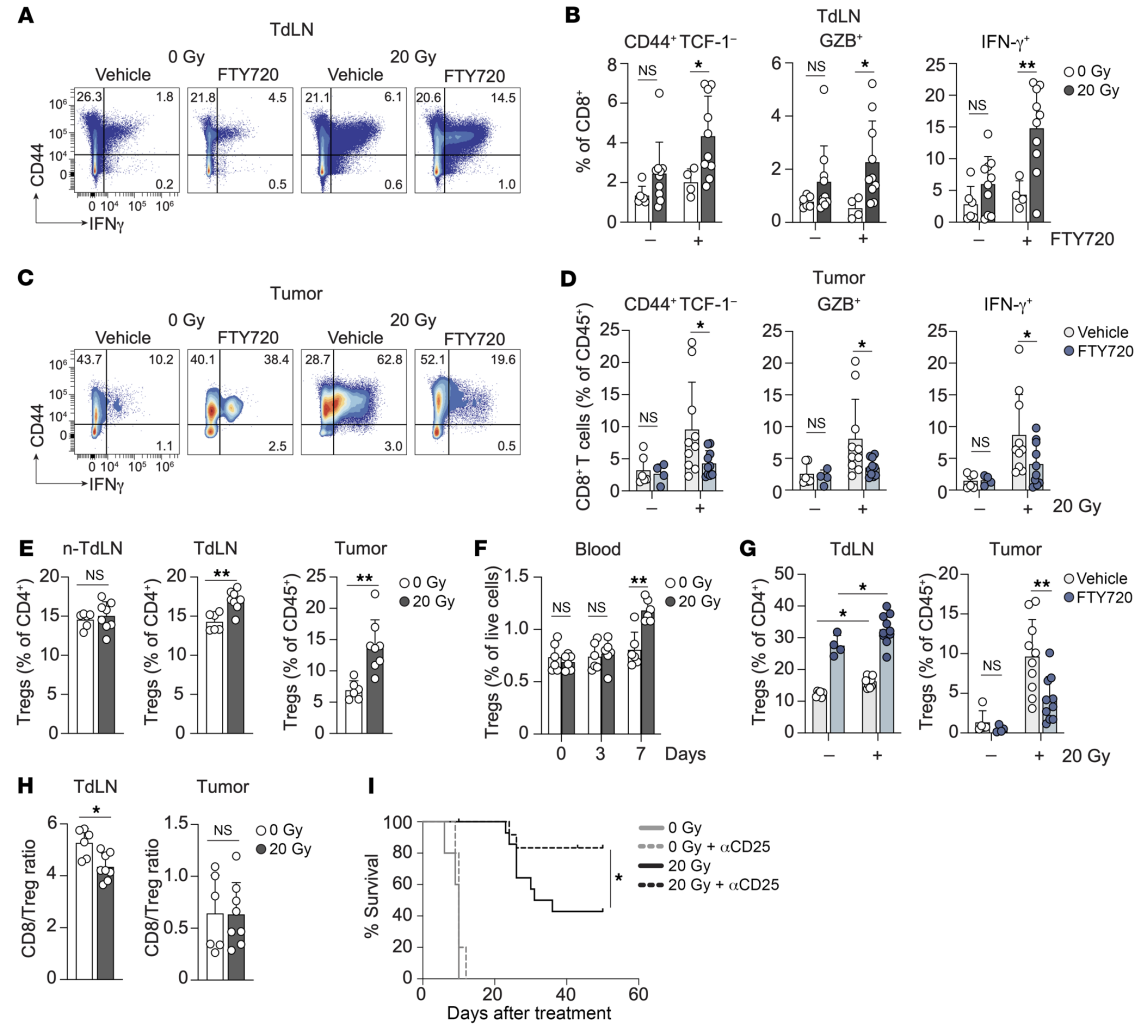
RT induces concomitant CTL and Treg responses in the TC-1 tumor model. (**A**–**D**) TC-1 tumor–bearing mice were treated with 20 Gy RT (*n* = 10) or control (0 Gy, *n* = 4–6) when tumors reached approximately 20 mm^2^ in size (day 0). FTY720 or vehicle (NaCl) was administered orally on days –1, 3, and 5. On day 8, the CD8^+^ T cell response was analyzed by flow cytometry in the TdLN (**A** and **B**) and tumor (**C** and **D**). (**A** and **C**) Representative concatenated flow cytometric plots showing IFN-γ^+^ cells among CD8^+^ T cells in the TdLN (**A**) and tumor (**C**). (**B** and **D**) Frequency of CD44^+^TCF-1^–^, GZB^+^, and IFN-γ^+^ cells among CD8^+^ T cells in the TdLN (**B**) and tumor (**D**). IFN-γ was measured after in vitro PMA/Ionomycin stimulation. (**E** and **F**) Monitoring of the (FOXP3^+^CD25^+^) Treg response to 20 Gy RT (*n* = 6–8) or control (0 Gy, *n* = 6) in TC-1 tumor–bearing mice on day 8 after treatment. (**E**) Treg frequency among CD4^+^ T cells in the non-TdLN and TdLN, or among CD45^+^ cells within the tumor. (**F**) Percentage of Tregs among live cells in blood at the indicated time points (*n* = 6/group). (**G**) Frequency of Tregs in the indicated tissues on day 8 following 20 Gy RT (*n* = 10) or control (0 Gy, *n* = 4–6) with or without FTY720 treatment. (**H**) CD8^+^ T cell/Treg ratio in the TdLN and tumor after RT. (**I**) OS of TC-1 tumor–bearing mice treated with 0 Gy (*n* = 5) or 20 Gy (*n* = 11–14/group) RT in combination with a CD25-depleting mAb or vehicle (PBS) administered i.p. on day –1 and on day 5 after RT. *P < 0.05 (Mantel-Cox analysis). Data are from 1 experiment and are representative of at least 2 experiments. Error bars indicate the SD. *P < 0.05 and **P < 0.01, by 2-way ANOVA with Bonferroni’s post hoc test (**B**, **D**, **F** and **G**) and Mann-Whitney *U* test (**E** and **H**).

The influx of effector CTLs in the irradiated TC-1 tumor probably originated from the induction of a de novo CD8^+^ T cell response in the TdLN by RT (15). In certain immunogenic mouse models, T cell priming proved important for durable RT-induced antitumor immunity (12, 13). To visualize new T cell priming after RT of the TC-1 tumor, mice were treated with the S1P receptor agonist FTY720, which traps T cells in LNs (42). This enlarges the window to identify newly primed T cells in the TdLN. We confirmed the efficacy of FTY720 efficacy by the elimination of circulating CD8^+^ and CD4^+^ T cells in peripheral blood (Supplemental Figure 4A). We found that treatment with FTY720 did not affect tumor development (Supplemental Figure 4B). At day 8 after RT, we analyzed T cell priming and effector differentiation in the TdLN. The flow cytometry panel included the transcription factor TCF-1 to monitor CTL effector differentiation (43). TCF-1 loss signifies reduced “stemness” (44) and a shift toward more differentiated effector T cells (43). In the presence of FTY720, a significant RT-induced increase in effector phenotype CD44^+^TCF-1^–^–, GZB^+^-, and IFN-γ^+^–expressing CD8^+^ T cells was revealed (Figure 3, A and B), whereas the effect of RT on effector phenotype CD4^+^ T cells was less pronounced (Supplemental Figure 4C). Moreover, FTY720 treatment revealed that a large part of the effector CD8^+^ T cells present in the tumor after RT originated from the TdLN, since their frequency in the tumor was significantly reduced upon FTY720 treatment (Figure 3, C and D). This was not evident for effector CD4^+^ T cells (Supplemental Figure 4D). Thus, in the lymphocyte-depleted TC-1 tumor model, RT elicited priming of CD8^+^ T cells that subsequently migrated into the irradiated tumor.

Despite RT-induced CTL priming, not all TC-1 tumor–bearing mice were cured (Figure 1D). Since the TC-1 tumor induced Treg priming during its development and because of the described increase in Tregs in the TME upon RT (28, 45, 46), we considered that RT might enhance the Treg response in the TC-1 tumor setting. Tregs reportedly require antigen-dependent activation and expansion in the TdLN prior to migration to a tumor (41, 47). Treg frequencies (Figure 3E and Supplemental Figure 4E) and absolute numbers (Supplemental Figure 4F) were significantly increased in the TdLN and tumor, but not in the non-TdLN, on post-RT day 8. In addition, overall Treg frequency was increased in blood over time (Figure 3F), and the frequency of proliferating (Ki67^+^) Tregs was enhanced in the TdLN but not in the non-TdLN following RT (Supplemental Figure 4, G and H). In contrast, the frequency of proliferating Tregs in the TME was significantly decreased upon RT (Supplemental Figure 4, G and H). These data suggest that RT induced Treg priming in the TdLN, followed by migration of these cells into the irradiated TME, rather than inducing Treg expansion in the TME (45). FTY720 treatment supported this observation, since the frequency of Tregs was increased in the TdLN after RT, while their frequency in the TME was decreased (Figure 3G). FTY720 treatment also led to an increase in Treg frequency in the TdLN of control mice (0 Gy). These data show that TC-1 tumor development induced priming and tumor infiltration by Tregs and that this was exacerbated by RT.

Thus, RT promoted the Treg response and also induced a new CD8^+^ T cell response, which significantly lowered the CD8^+^ T cell/Treg ratio in the TdLN and maintained the unfavorable CD8^+^ T cell/Treg ratio in the tumor (Figure 3H) . Therefore, Tregs might be an impediment to CTL-mediated tumor control upon RT. To test this, we treated mice with an Fc-modified antibody against CD25 (48) that efficiently depleted peripheral and intratumoral Tregs (Supplemental Figure 5, A and B) but not CD8^+^ or CD4^+^ Tconvs (Supplemental Figure 5C), both before and after RT (Supplemental Figure 5D). This treatment greatly improved TC-1 tumor control and OS (Figure 3I and Supplemental Figure 5E). A 3× 8 Gy RT regimen gave similar results (Supplemental Figure 5, F and G). Taken together, these data indicate that in the TC-1 tumor model, Tregs limited RT-mediated tumor eradication, probably by inhibiting the RT-induced CTL response.

### CTLA-4 blockade increases the RT-induced Treg response and does not improve tumor control.

**Figure 4:**
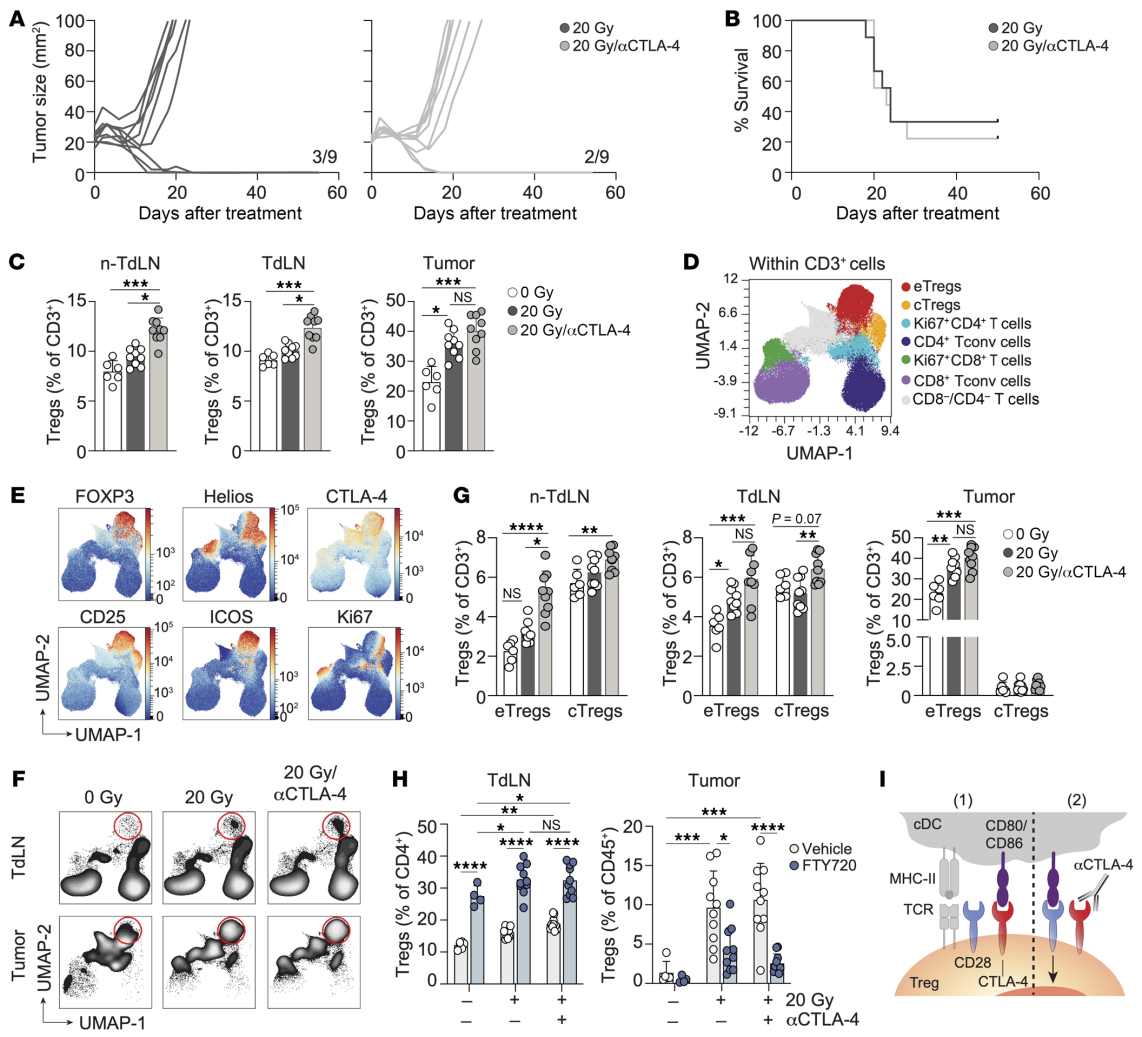
CTLA-4 blockade exacerbates RT-induced eTreg expansion. Mice bearing 20 mm^2^ TC-1 tumors received RT (20 Gy, *n* = 9) or control (0 Gy, *n* = 6) on day 0. Treatment included vehicle (PBS) or a CTLA-4–blocking mAb on days 0, 3, 6, and 9, with longitudinal monitoring (**A** and **B**) and flow cytometric analysis of the non-TdLN, TdLN, and tumor on post-treatment day 8 (**C**–**G**). (**A**) Individual tumor growth curves and (**B**) OS for the treatment groups. Ratios indicate the number of mice that showed full recovery upon treatment compared with the total. (**C**) Percentage of total Tregs among CD3^+^ lymphocytes in the indicated tissues on day 8. (**D**–**F**) UMAP display of 2,500 randomly selected CD3^+^ T cells per sample in non-TdLN, TdLN, and tumor on day 8 for all treatment groups combined, with FlowSOM-guided clustering (see also Supplemental Figure 2B) (**D**) and marker visualization (**E**) used to highlight the eTreg response. (**F**) UMAP visualization of the response of the CD3^+^ T cell subpopulations in the TdLN and tumor to the indicated treatments. Red circles highlight the eTreg population. (**G**) Frequencies of eTregs and cTregs identified in **D** among CD3^+^ T cells found in the indicated tissues on post-treatment day 8. (**H**) TC-1 tumor–bearing mice received 20 Gy (*n* = 10/group) or control (0 Gy, *n* = 4–6), with CTLA-4 mAb blockade or vehicle on days 0, 3, and 6, with or without FTY720. Treg frequencies were measured in the TdLN and tumor on post-RT day 8 (same experiment as in Figure 3G). (**I**) Visual representation of how Tregs benefit from CTLA-4 blockade. Data are from 1 experiment and are representative of 2 experiments. Error bars indicate the SD. *P < 0.05, **P < 0.01, ***P < 0.001, and ****P < 0.0001, by Kruskal-Wallis with Dunn’s post hoc test (**C** and **G**) and 2-way ANOVA with Tukey’s multiple-comparison test (**H**).

CTLA-4 blockade has been shown to enhance RT-induced tumor regression in mouse models (49, 50) and clinical studies (22, 23, 51). To evaluate the effects of CTLA-4 blockade in our lymphocyte-depleted TC-1 tumor model, we treated tumors with RT and either vehicle or a blocking antibody against CTLA-4 that does not deplete Tregs (52, 53) on successive days. Anti–CTLA-4 treatment did not improve RT-induced TC-1 tumor control or OS (Figure 4, A and B). Interestingly, CTLA-4 blockade increased the RT-induced Treg response in both TdLNs and non-TdLNs, and the Treg population remained high in the tumor (Figure 4C). The majority of these Tregs expressed Helios (Supplemental Figure 6, A and B), indicating that RT and CTLA-4 blockade promoted the response of thymus-derived Tregs. To more comprehensively characterize how CTLA-4 blockade affected the T cell response, we performed FlowSOM-guided clustering analysis and dimensionality reduction on the CD3^+^ T cell populations in the different tissues (Figure 4, D and E, and Supplemental Figure 6C). CTLA-4 blockade in the context of RT significantly increased the frequencies of both eTregs and cTregs in the non-TdLN and TdLN, as compared with RT alone (Figure 4, F and G). RT as a single treatment selectively increased the proportion of eTregs, but not of cTregs, in the TdLN (Figure 4G), suggesting that RT was required to facilitate cTreg-to-eTreg conversion. In the tumor, RT alone and in combination with CTLA-4 blockade increased eTreg frequencies (Figure 4G). FTY720 treatment revealed that CTLA-4 blockade supported RT-induced Treg expansion in the TdLN, rather than inducing Treg expansion in the TME (Figure 4H and Supplemental Figure 6D) (53). Specifically, Tregs migrated from the TdLN to the tumor, as shown by the strong reduction in Treg frequencies in the tumor after FTY720 treatment.

Thus, the TC-1 tumor promoted priming of thymus-derived Tregs in the TdLN, and RT, alone and in combination with CTLA-4 blockade, further supported this process. Subsequently, these newly primed Tregs populated the tumor. Tregs are highly dependent on CD28 costimulation for their expansion (54, 55). Given the prevalence of Tregs in the TdLN of the TC-1 tumor, CTLA-4 blockade may favor Treg over Tconv responses. Tregs may capitalize on the increased availability of CD80 and/or CD86 on cDCs following CTLA-4 blockade, leading to enhanced CD28 costimulation and subsequent Treg priming (Figure 4I).

### CD86, rather than CD80, promotes the RT-induced Treg responses.

**Figure 5:**
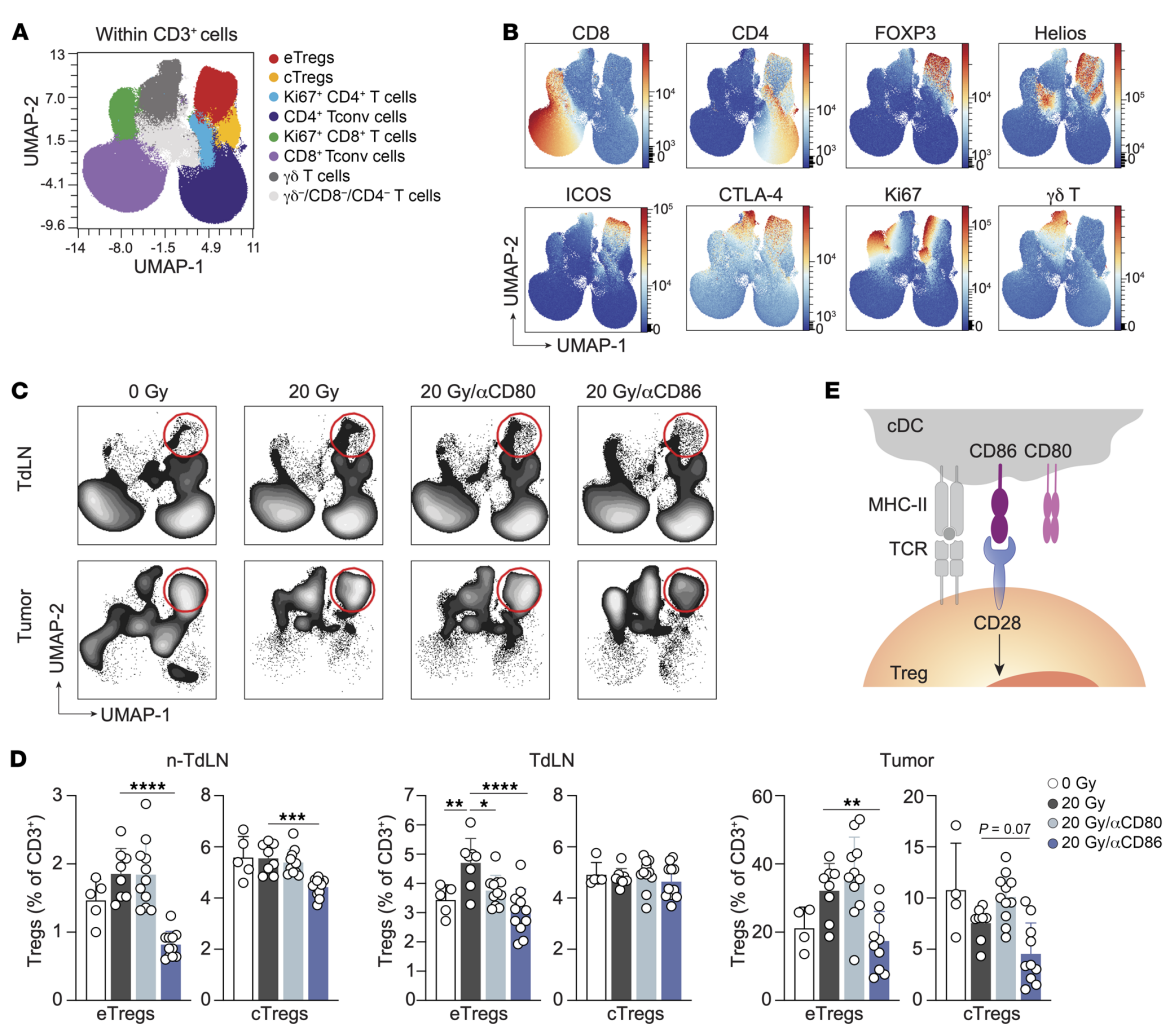
CD86, but not CD80, drives the RT-induced eTreg response. Mice bearing 20 mm^2^ TC-1 tumors received control treatment (0 Gy, *n* = 5) or 20 Gy RT on day 0 in combination with either vehicle (PBS, *n* = 8) or a blocking mAb against CD80 (*n* = 11) or CD86 (*n* = 11) on days 0, 3 and 6. The CD3^+^ lymphocyte response was monitored by flow cytometry in the non-TdLN, TdLN, and tumor on day 8. (**A**–**C**) UMAP visualization of 2,500 randomly selected CD3^+^ cells per sample found in the non-TdLN, TdLN, and tumors on day 8 of all treatment groups combined. FlowSOM-guided clustering (**A**) identifying the same cell populations as found in the previous figures and (**B**) representative heatmaps of the markers included to determine the CD3^+^ T cell subpopulations. (**C**) Visualization of the response of the CD3^+^ T cell subpopulations in the TdLN and tumor to the indicated treatments. Red circles highlight the eTreg population. (**D**) Frequencies of eTregs and cTregs identified in **B** among CD3^+^ cells found in the indicated tissues on post-treatment day 8. (**E**) Graphic visualization of how CD86, but not CD80, binds CD28 to support Treg expansion. Data are from 1 experiment and are representative of 2 experiments. Error bars indicate the SD. *P < 0.05, **P < 0.01, ***P < 0.001, and ****P < 0.0001, by ordinary 1-way ANOVA with Dunnett’s post hoc test (**D**).

The findings above highlight the importance of the CD28 costimulatory axis in regulating Treg expansion. They raise the possibility that the CD28 ligands CD80 and/or CD86 may dictate Treg numbers after RT in the TC-1 tumor model. We therefore selectively blocked CD80 or CD86 in the presence of RT and examined the T cell response in detail by spectral flow cytometry as before (Figure 5, A and B). Interestingly, we observed that CD86 blockade significantly reduced the RT-induced eTreg population in the non-TdLN, TdLN, and tumor (Figure 5, C and D). After CD86 blockade, the frequencies of eTregs in these tissues were comparable to those in nonirradiated (0 Gy) mice. CD86 blockade diminished the proportion of cTregs to some extent in the non-TdLN but not in the TdLN. In contrast, CD80 blockade in the context of RT only reduced the frequency of eTregs in the TdLN (Figure 5, C and D). Thus, in the TC-1 tumor setting, CD86 is the selective CD28 ligand that supports the generation of an eTreg response after RT (Figure 5E).

### CD86 blockade in the context of RT improves cDC costimulatory status and CTL priming.

**Figure 6:**
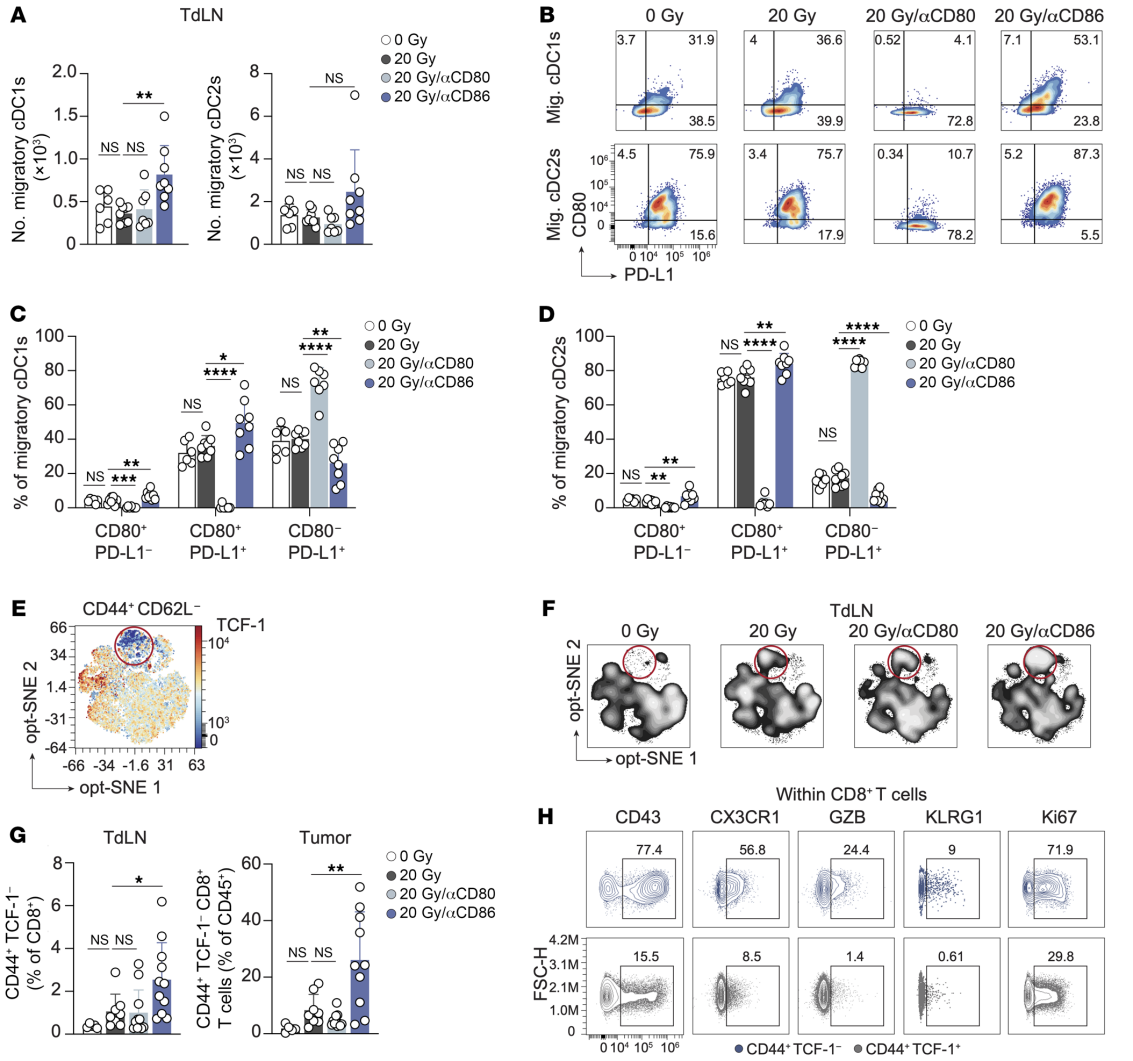
CD86 blockade in the context of RT improves cDC costimulatory status and CTL priming. (**A**–**D**) Mice bearing 20 mm^2^ TC-1 tumors received 0 Gy (*n* = 6) or 20 Gy RT on day 0 in combination with either vehicle (PBS, *n* = 8) or a blocking mAb against CD80 (*n* = 7) or CD86 (*n* = 8) on days 0, 3, and 6. The cDC response was monitored by flow cytometry in the TdLN on day 8. (**A**) Absolute counts of migratory cDCs1 and cDC2s. (**B**) Representative concatenated (*n* = 6–8) flow cytometric plots depicting the percentage of CD80^+^ and/or PD-L1^+^ T cells among migratory (Mig.) cDC1s and cDC2s in the TdLN per treatment group. The numbers in the boxes indicate percentages. (**C** and **D**) Quantification of the cell populations represented in **B** among migratory cDC1s (**C**) and migratory cDC2s (**D**) from the TdLN. (**E**–**H**) The CD8^+^ T cell response was monitored by flow cytometry in the same experiment described in Figure 5. (**E** and **F**) Opt-SNE visualization of 1,000 randomly selected CD44^+^CD62L^–^ cells among CD8^+^ T cells per sample found in TdLNs on day 8, concatenated per treatment group. (**E**) Representative heatmap of TCF-1 expression and (**F**) visualization of the TCF-1^–^ subpopulation in the TdLN (encircled) in different treatment groups. (**G**) Frequency of CD44^+^TCF-1^–^ cells among CD8^+^ T cells found in the TdLN and among CD45^+^ cells in the tumor on post-treatment day 8. (**H**) Concatenated (*n* = 11) contour plots depicting expression of the indicated markers on CD44^+^TCF-1^–^ cells and CD44^+^TCF-1^+^ cells within CD8^+^ T cells in the TdLN. Numbers indicate percentages. Data are from 1 experiment and are representative of 2 experiments. Error bars indicate the SD. *P < 0.05, **P < 0.01, and ****P < 0.0001, by ordinary 1-way ANOVA with Dunnett’s post hoc test (**A** and **C**–**E**).

To clarify how CD80/CD86 blockade affected T cell priming, we performed flow cytometry to examine migratory cDC1s and cDC2s, which are responsible for T cell priming (56–58) (Supplemental Figure 7A). The absolute number of cDC1s or cDC2s in the TdLN remained unchanged with RT alone as compared with the control. However, the combination of RT and CD86 blockade significantly increased cDC1 numbers, and we observed a similar trend for cDC2s (Figure 6A). CD86 is constitutively expressed on cDCs, while CD80 is upregulated upon activation (29). In the context of RT, CD86 blockade significantly increased CD80 expression on both cDC1s and cDC2s, whereas CD86 expression remained unaffected (Supplemental Figure 7, B and C). CD86 and CD80 blockade had no significant effect on CD40 or PD-L1 expression on either cDC1s or cDC2s (Supplemental Figure 7, B and C).

On cDCs, CD80 can form a heterodimer with PD-L1. This CD80:PD-L1 heterodimer engages CD28, but cannot bind to PD-1, nor can it be downregulated by CTLA-4 (59, 60). Coexpression of CD80 and PD-L1 on cDCs positively correlated with enhanced CTL priming capacity against cancer, in agreement with increased formation of a CD28-costimulatory CD80:PD-L1 heterodimer (61). In the TC-1 tumor model, the frequency of cDC1s and cDC2s coexpressing CD80 and PD-L1 was significantly increased when RT was combined with CD86 blockade (Figure 6, B–D). The frequency of CD80^+^PD-L1^–^ cells was also increased, whereas the frequency of CD80^–^PD-L1^+^ cells was decreased. Thus, in the TC-1 model, RT-induced CTL priming was likely increased upon CD86 blockade by increasing the frequency and costimulatory state of migratory cDC1s that present tumor antigen in the TdLN.

To study CTL priming, we performed optimized stochastic neighbor embedding (opt-SNE) analysis of CD8^+^ T cells with a CD44^+^ CD62L^–^ effector phenotype found in the TdLN. Contour plot visualization revealed that the TCF-1^–^ subpopulation among CD44^+^CD62L^–^ cells in the TdLN was enlarged after RT and further increased upon combined treatment with CD86, but not CD80, blockade (Figure 6, E and F). Manual gating (Supplemental Figure 7D) confirmed these findings and showed that CD86 blockade in the context of RT increased the frequency of CD44^+^TCF-1^–^ cells among CD8^+^ T cells in both the TdLN and tumor (Figure 6G). Phenotypical analysis also showed increased expression of the effector differentiation markers CD43, CX3CR1, GZB, and KLRG1 on the CD44^+^TCF-1^–^CD8^+^ T cell population as compared with the CD44^+^TCF-1^+^ T cell population (Figure 6H). Moreover, the frequency of Ki67^+^CD44^+^TCF-1^–^ T cells was increased, indicating increased cell-cycle activity (Figure 6H). The collective findings indicate that CD86 blockade improved RT-induced CTL priming, expansion, and effector differentiation, which are likely facilitated by an increased presence of costimulatory migratory cDC1s in the TdLN.

### RT plus PD-1 blockade increases the Treg response, which is overruled by CD86 blockade, resulting in improved tumor control.

**Figure 7:**
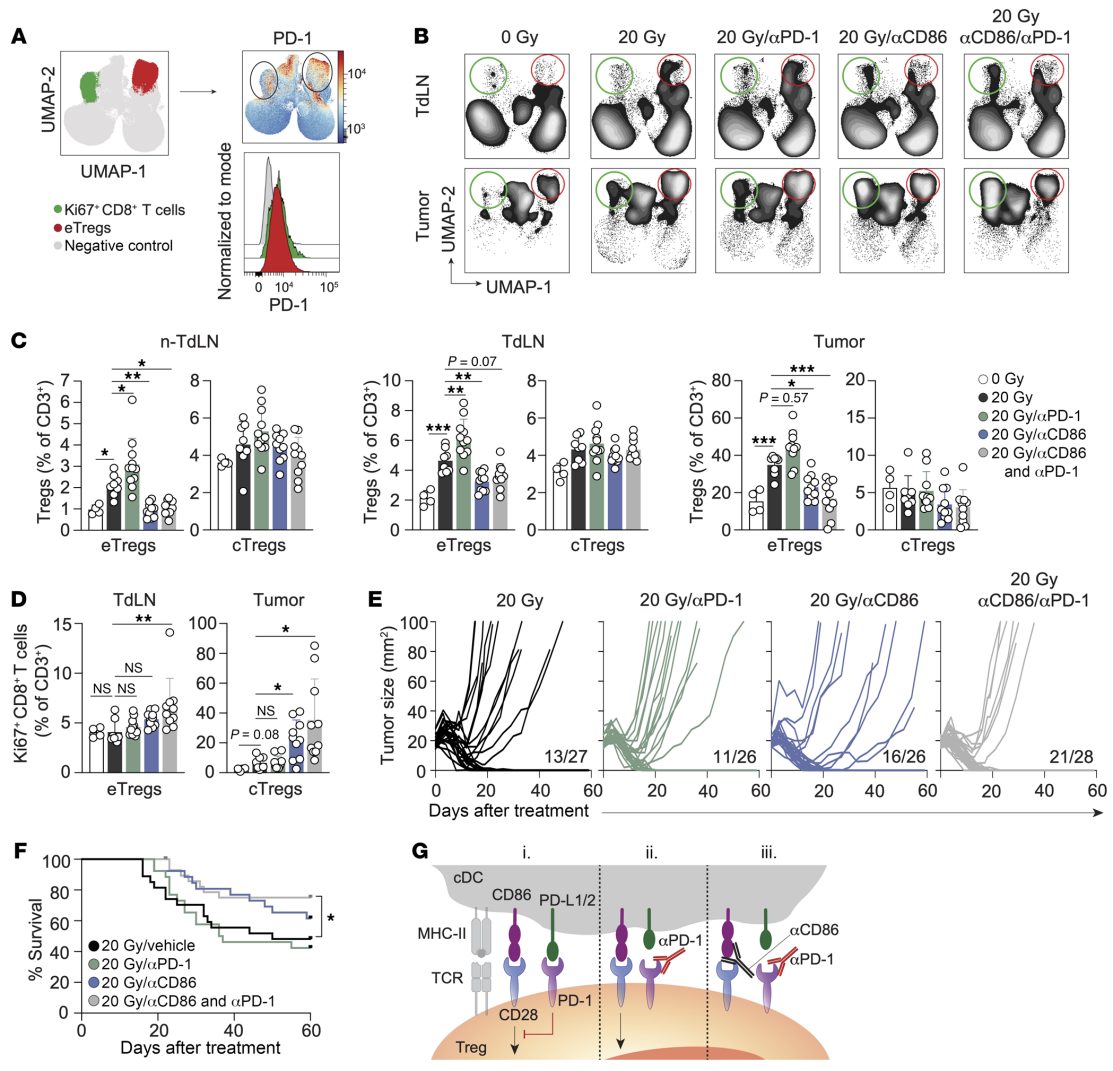
CD86-mediated CD28 costimulation is required for PD-1–dependent eTreg expansion. (**A**) PD-1 expression on Ki67^+^CD8^+^ T cells (green) and eTregs (red) in the tumor as identified in Figure 5A, presented as a heatmap and a representative histogram across all experimental conditions. (**B**–**D**) TC-1 tumor–bearing mice received 0 Gy (*n* = 4) or 20 Gy RT on day 0 with vehicle (PBS, *n* = 8) or blocking mAbs against PD-1 (*n* = 11), CD86 (*n* = 10), or their combination (*n* = 10) on days 0, 3, and 6. CD3^+^ lymphocyte responses were analyzed by flow cytometry in non-TdLNs, TdLNs, and tumor on day 8. (**B**) UMAP visualization of the treatment response of the CD3^+^ T cell subpopulations. The red circle indicates eTregs, and the green circle indicates Ki67^+^CD8^+^ T cells (see also Supplemental Figure 8, B and C). (**C**) Frequencies of eTregs and cTregs identified in Supplemental Figure 8B among CD3^+^ T cells in the indicated tissues. (**D**) Quantification of the Ki67^+^CD8^+^ T cell population among total CD3^+^ T cells in the TdLN and tumor. (**E**) Individual tumor growth curves and (**F**) OS of TC-1 tumor–bearing mice receiving RT on day 0 with vehicle (*n* = 27), blocking mAbs against PD-1 (*n* = 26), CD86 (*n* = 26), or a combination (*n* = 28) on days 0, 3, and 6. Proportion of mice that fully recovered is indicated. (**G**) Proposed effect of combined CD86 and PD-1 blockade on Tregs. (i) PD-L1/L2 on cDCs engages PD-1, which inhibits CD28 costimulation of Tregs. (ii) PD-1 blockade enables CD28 costimulation of Tregs. (iii) CD86 blockade inhibits CD28 costimulation of Tregs, which cannot be overruled by PD-1 blockade, impeding the Treg response. Data are from 1 experiment and are representative of 2 experiments. Error bars indicate the SD. *P < 0.05, **P < 0.01, and ***P < 0.001, by ordinary 1-way ANOVA with Dunnett’s post hoc test (**C**), Brown-Forsythe ANOVA with Dunnett’s T3 post hoc analysis (**D**), and Mantel-Cox analysis (**F**).

PD-1 is the key target in cancer immunotherapy, and its expression is considered a hallmark of suboptimally primed CTLs that lack full cytotoxic effector functions (62). We found that CD44^+^TCF-1^–^CD8^+^ T cells in the tumor after combined RT and CD86 blockade expressed PD-1, albeit to a lesser extent than did CD44^+^TCF-1^+^CD8^+^ T cells (Supplemental Figure 8A). In fact, both Ki67^+^ CTLs and eTregs in the tumor expressed PD-1 (Figure 7A). PD-1 preferentially inhibits CD28 costimulation (31), and PD-1 blockade not only promotes Tconv responses (2) but also Treg responses by enabling TCR/CD28 signaling (63, 64). We therefore examined the effect of PD-1 blockade alone, or in combination with CD86 blockade on RT-induced Treg and CTL responses. Strikingly, we found that PD-1 blockade increased RT-induced eTreg priming and tumor infiltration (Figure 7, B and C, and Supplemental Figure 8, B and C). This result argues that CD28 costimulation, enabled by PD-1 blockade, favored Treg priming in this tumor model, as did CTLA-4 blockade. Upon CD86 blockade, the RT-induced eTreg response was abrogated both in the absence and the presence of PD-1 blockade (Figure 7, B and C). These data indicate that CD86 was required to engage CD28 on Tregs to drive their response. Importantly, following CD86 blockade, the frequency of proliferating (Ki67^+^) CD8^+^ T cells significantly increased in the tumor, while combined CD86 and PD-1 blockade increased this cell population in both the TdLN and tumor (Figure 7, B and D, and Supplemental Figure 8, B and C). These findings align with our initial observation that RT-induced Treg priming hampered the induction of a CTL response by RT.

We next assessed how inhibition of PD-1 and/or CD86 affected RT-induced tumor control. PD-1 blockade alone failed to enhance RT-induced tumor regression and OS, in line with stimulation of the Treg response (Figure 7, E and F, and Supplemental Figure 8D). CD86 blockade alone improved RT-induced tumor control from 48% to 62%, with a fraction of the tumors initially responding, but later relapsing. Combined PD-1 and CD86 blockade increased OS compared with RT alone. The effect of CD86 blockade alone on overall mouse survival revealed a similar trend, but did not reach statistical significance. Taken together, in this lymphocyte-depleted tumor model, RT enhanced eTreg priming while restraining tumor-reactive CTL priming, which was further enhanced by PD-1 blockade. This was likely because PD-1 blockade preferentially enabled CD28 costimulation of Tregs (Figure 7G). CD86 blockade alone, or in combination with PD-1 blockade, counteracted eTreg priming through the inhibition of CD28 costimulation of Tregs. Inhibition of the Treg response facilitated tumor-reactive CTL priming and tumor control by RT upon CD86 blockade. With additional PD-1 blockade, PD-1^+^ CTLs likely exhibit enhanced activity in the TME, explaining the improved tumor control.

## Discussion

The potential of RT to induce systemic T cell responses to cancer has recently received much attention, but clinical evidence for abscopal, immune-mediated effects is scarce, even in combination with ICB (10). We must therefore better understand the ability of RT to induce tumor-controlling T cell responses in the context of immunologically divergent cancer types. Comparison of in vivo tumor models of varying immunogenicity demonstrated that in immunogenic tumors, intratumoral CD8^+^ T cells contributed to the RT response. In poorly immunogenic tumors, however, RT fails to elicit a systemic antitumor immune response or an abscopal effect (20). We show that the TC-1 tumor model used in our study recapitulated lymphocyte-depleted human cancer types (3) that respond negatively to RT (Figure 1A). The TC-1 tumor expresses HPV-16–derived E6 and E7 antigens but is not immunogenic and only regresses upon therapeutic vaccination (37). We show that this tumor invited Tregs into the tumor and the TdLN, consistent with the systemic immunosuppression clinically observed in this tumor type (65, 66). Priming tumor-specific CTLs in the TdLN depends on cDC1s that excel at tumor antigen cross-presentation (56, 57). The cDC2 subset, including Tconvs and Tregs, favors CD4^+^ T cell priming (58, 67). In the TC-1 tumor, the cDC2 frequency far exceeded the frequency of cDC1s (Figure 2A), which may have favored Treg priming. Nevertheless, RT-induced TC-1 tumor regression was CD8^+^ T cell dependent. This finding suggests that in lymphocyte-depleted tumors, there is an unexploited, favorable CTL response that should be improved by the correct intervention(s).

Tregs serve to prevent or suppress unwanted Tconv responses against both self- and foreign antigens (68). At steady state, “immature” or “tolerogenic” cDCs that mainly express CD86 as a costimulatory ligand (69) migrate from peripheral tissues to dLNs to present self-antigens and prevent responses of sporadic, autoreactive T cells. FOXP3^+^Helios^+^ thymus-derived cTregs have this effect at steady state without clonal expansion or relocation to nonlymphoid tissues. Reportedly, the metabolic state of cDC2s may govern cTreg expansion, in part through CD86 upregulation (70). Especially in tumors, limited nutrient resources and immunosuppressive factors may induce a metabolic state in cDCs that supports Treg expansion (71). Tregs help to control inflammation resulting from tissue injury, such as that inflicted by RT. In this process, cTregs are recruited from dLNs to damaged tissues (72), where they present an eTreg phenotype (39). The stimuli that drive the eTreg response are currently unknown. Murine and human tissue–resident eTregs have a conserved transcriptional signature that is most explicit in tumor-resident eTregs and contains a tissue repair program (73). In an irradiated tumor, next to extinguishing inflammation, these eTregs support extracellular matrix remodeling and tumor growth via their repair function (74) and may thereby impede RT efficacy.

We document that the TC-1 tumor at steady state drives the priming of Tregs in the TdLN that expand and populate the tumor. These cells coexpress FOXP3 and Helios, indicating their tTreg identity. We found that the Treg response in the TC-1 tumor model was enforced by RT and further promoted by CTLA-4 blockade. Tregs constitutively express CTLA-4 that inhibits CD28 costimulation. Upon CTLA-4 blockade, both CD80 and CD86 are available to support the Treg response by CD28 costimulation (53). However, we found that CD86 was the selective driver of this Treg response. This aligns with recent in vitro studies highlighting CD86 as the preferred ligand for driving CD28 costimulation of Tregs (75). These authors attributed this preference to the constitutive presence of CTLA-4 on Tregs. Since CD86 exhibits a lower affinity for CTLA-4 than does CD80, Tregs predominantly rely on CD86 for CD28 costimulation (75). We found that CD86, but not CD80, blockade led to increased migratory cDC1 frequencies in the TdLN, along with coordinated CD80 and PD-L1 expression that favored the formation of a CD28-costimulatory CD80:PD-L1 heterodimer. These data suggest that Tregs in the TME constrained the migratory and costimulatory properties of cDC1s, thereby limiting CTL priming in the TdLN. Furthermore, as documented in other tumor settings (76), Treg accumulation in the TdLN can restrict CTL priming by inhibiting cDC1 activation.

In certain mouse tumor models (TS/A and 4T1 breast cancer and MCA38 colon cancer), CTLA-4 blockade and RT have a combined therapeutic effect (23, 38, 49, 50). CTLA-4 likely promotes new T cell priming in these models, given the observed increase in TCR diversity of tumor-infiltrating T cells. Subsets of patients with metastatic non–small cell lung cancer (23) or metastatic melanoma (22) also showed a combined effect of CTLA-4 blockade and RT. This was not the case in the TC-1 model, which we explain by increased Treg over CTL priming. CTLA-4 blockade efficacy is known to largely rely on a high CTL over Treg ratio in the tumor (77, 78). In T cell–devoid tumors, several factors work against a favorable CTL/Treg ratio, e.g., a higher cDC2/cDC1 ratio in the TME, limited RT-induced adjuvanticity (27), and/or RT-induced suppressive factors that prevent cDC1 maturation (79, 80). Reportedly, fractionated low-dose RT is superior in eliciting IFN-I–dependent optimization of cDC1s for CTL priming because single high-dose RT attenuates IFN-I release by promoting DNA degradation (38). Consequently, 3× 8 Gy, but not 20 Gy, cooperated with CTLA-4 blockade to improve systemic antitumor immunity in TS/A and 4T1 mouse models (23, 38, 49, 50). However, in our model, RT induced a strong Treg response to both 3× 8 Gy and 20 Gy, and these schedules had no differential therapeutic effect. Thus, in Treg-dominant tumors, CTLA-4 blockade may preferentially support Treg expansion (81) and not improve CTL-based tumor control, regardless of the RT regimen used (46, 82).

In the TC-1 tumor setting, PD-1 blockade exacerbated the RT-induced eTreg response and, consequently, impeded the therapeutic CTL response. In agreement with this, PD-1 blockade was recently shown to promote Treg responses in certain patients with cancer, potentially leading to cancer hyperprogression (63, 83). These studies showed that both Tregs and Tconvs can profit from CD28 costimulation that is enabled by PD-1 blockade (31). In tumors that favor Treg over CTL priming at steady state and display an exacerbated eTreg response upon RT, the conditions are met for further Treg priming and expansion upon PD-1 blockade. Our discovery that CD86 blockade abrogated the Treg response in this setting is therefore of potential clinical relevance. When CD86 was blocked, PD-1 blockade could not induce Treg expansion upon RT, indicating its dependence on CD86-mediated CD28 costimulation. Importantly, RT-induced CTL priming supported by CD86 blockade allowed for reversal of the Treg/CTL ratio, whereas PD-1 blockade likely improved CTL quality, thereby enhancing tumor control. Our finding that CD86 blockade primarily inhibited the eTreg response is of interest, since it is advisable to avoid interference of ICB with peripheral tolerance induction by cTregs to prevent adverse immune-related toxicities (84).

In conclusion, in a model of lymphocyte-depleted cancer that favors myeloid and Treg infiltration, we reveal that CTLA-4 and PD-1 blockade had the opposite effect on RT-induced tumor control compared with immunogenic tumors with high Tconv infiltrates. This was due to exacerbation of RT-induced Treg responses that counteracted the RT-induced CTL response. We therefore caution that CTLA-4 and/or PD-(L)1 blockade may likewise exacerbate RT-induced Treg responses in human lymphocyte–depleted cancer. Our findings argue that CD86 is a suitable target to inhibit undesired eTreg responses and a potential new candidate to improve Tconv responses to poorly immunogenic cancers, particularly in combination with RT.

We acknowledge the limitation that our study was based on 1 murine tumor cell line representing lymphocyte-depleted cancer and that the immunological mechanisms revealed in our study should be corroborated in additional representative tumor models to validate the generality of our findings.

## Methods

### TCGA data analysis.

Immune subtype classifications among 9,126 tumors were collected from Thorsson et al. (3). Patient-specific RT status and survival metrics were gathered from the UCSC Xena Platform using the UCSCXenaTools package (85), in which complete information was available for 7,891 tumors. Kaplan-Meier curves were generated for each immune subtype using OS (in months) by RT status (yes vs. no). For immune subtype prediction, the C4/C5 subtypes were collapsed into a single immune subtype, and tumors derived from the C3 and C4/C5 immune subtypes were selected (*n* = 3,939). Features derived from the CIBERSORT deconvolution algorithm and IFN-γ signature were subsequently used (*n* = 23). Next, data were split into 70% and 30% training and testing data sets, respectively. The training data were scaled and centered before undergoing a 5-fold repeated cross-validation strategy to predict between C4/C5 and C3 using a KNN model. The test data were then applied to evaluate model performance.

### Murine microarray analysis.

Microarray data and metadata were downloaded from the Gene Expression Omnibus (GEO) database (GEO GSE85509) using GEOquery. Murine gene symbols were converted to human symbols using the biomaRt package. Immune cell types were deconvolved using CIBERSORT from the immunedeconv package, and the IFN-γ signature was generated using the Ayers gene signature (86). Next, the data from the TC-1 and MC38 cell lines were used as input into the trained KNN model for classification.

### Tumor cells.

The MC38 colon cancer cell line was purchased from Kerafast, and TC-1 tumor cells (lung epithelial cells engineered to express HPV16 E6 and E7 proteins; ref. 34) were obtained from Leiden University Medical Center in 2015 (the authors did not perform further authentication). MC38 and TC-1 cells were cultured in DMEM and RPMI 1640 (Gibco, Life Technologies, Thermo Fisher Scientific), respectively, supplemented with 10% FCS, 0.1 mM nonessential amino acids, 1 mM sodium pyruvate, 2 mM l-glutamine, 10 mM HEPES, and penicillin/streptomycin (Roche) at 37°C, 5% CO_2_. MC38 and TC-1 cell stocks were tested negative for mycoplasma by PCR, and thawed cells were used within 3 passages for in vivo experiments.

### Tumor transplantation and RT.

Six- to 8-week-old female C57BL/6Rj (B6) mice were purchased from Janvier Laboratories. On day –8, mice were anesthetized with isofluorane and injected s.c with either 1 × 10^6^ MC38 or 1 × 10^5^ TC-1 tumor cells in 50 μL HBSS. Tumor size was measured by calipers in 2 dimensions and calculated as follows: area (mm^2^) = width × length. RT was initiated when the tumors reached 18–25 mm^2^ (day 0) in size, and mice were randomly assigned to different treatment groups. RT was applied using the SmART^+^ system (Precision X-Ray). Mice were anesthetized with isoflurane, and a cone beam CT scan of the mice was performed. The tumor was localized on the CT scan and targeted with RT at 0.1 mm precision using round collimators of 1.0 or 1.5 cm in diameter. A single fraction of 8 or 20 Gy (225 peak kilovoltage [kVp]), filtered with 0.3 mm copper (3 Gy/min) was delivered. For fractionated dosage studies, a single dose of 8 Gy was delivered on days 0, 1, and 2. Control mice (indicated as 0 Gy) were anesthetized and subjected to a cone beam CT scan but were not exposed to RT. Mice were sacrificed when the tumor diameter reached 15 mm or when the tumor size reached greater than 100 mm^2^ in size. In the survival curves, censored events indicate mice that were sacrificed due to disease unrelated to the treatment.

### Therapeutic antibodies and reagents.

Mice received i.p. injections of depleting anti-CD8α mAbs (2.43, Bio X Cell) or anti-CD4 mAbs (GK1.5, Bio X Cell) at 200 μg per mouse in 100 μL PBS starting on day –1 prior to RT (day 0) and then on days 3, 6, and 9. For Treg depletion experiments, mice were injected i.p. with 250 μg depleting mouse IgG2a isotype CD25 mAbs (48) (modified clone of PC61, Evitria) in 100 μL PBS on day –1 prior to RT and on day 5. Blocking mAbs against CTLA-4 (UC10-4F10-11, Bio X Cell), PD-1 (RMP1-14, Bio X Cell), CD80 (1G10, Bio X Cell), and CD86 (GL-1, Bio X Cell) were injected i.p. at either 100 μg (anti–CTLA-4 and anti–PD-1) or 200 μg (anti-CD80 and anti-CD86) per mouse in 100 μL PBS on the day of RT (day 0) and on days 3 and 6 and, in the case of anti–CTLA-4, also on day 9. Control mice were injected with equal amounts of PBS (vehicle) according to the treatment schedule indicated. FTY720 (Fingolimod, Cayman Chemical) was dissolved in 0.9% NaCl solution (vehicle) and administered at 2 mg/kg by oral gavage. FTY720 treatment started 1 day prior to RT and was repeated 3 times per week throughout the duration of the experiment.

### Tissue preparation and flow cytometry.

At the indicated time points, tumor-bearing mice were sacrificed, and the lymphoid tissues and tumors were isolated. Intratumoral injection of 5% Evans Blue dye (MilliporeSigma) in 50 μL PBS identified the axillary LN on the same tumor-bearing side as the TdLN, whereas the contralateral inguinal LN was defined as the non-TdLN. The TdLN was carefully kept out of the field of irradiation to prevent RT-induced attenuation of the adaptive immune responses in the LN (87). Tumor tissue was mechanically disaggregated using a McIlwain tissue chopper (Mickle Laboratory Engineering), and a single-cell suspension was prepared by digesting the tissue in collagenase type A (Roche) and 25 μg/mL DNase I (MilliporeSigma) in serum-free DMEM for 45 minutes at 37°C. Enzyme activity was neutralized by adding medium with 10% FCS, and the tissue was dispersed by passing through a 70 μm cell strainer. To acquire single-cell suspensions, LN tissue was punctured with a 27 gauge needle followed by incubation in 100 μg/mL Liberase TL (Roche) in serum-free DMEM for 30 minutes at 37°C. Enzyme activity was neutralized as described above, and tissue was dispersed by passing it through a 70 μm cell strainer. Microvette CB300 LH tubes (Sarstedt) were used to collect peripheral blood cells from the tails of live mice. RBCs were lysed in 0.14 M NH_4_Cl and 0.017 M Tris-HCl (pH 7.2) for 1 minute at room temperature. For surface staining, single cells from the isolated tissues (except blood samples) were first incubated for 10 minutes on ice with anti-CD16/CD32 (1:50, clone 2.4G2, BD Bioscience) supplemented with 10 μg/mL DNAse to block nonspecific Fc receptor binding. Next, surface antibody staining was performed (Supplemental Table 1) for 30 minutes in PBS containing 0.5% BSA and 0.01% sodium azide. For intracellular staining of transcription factors and cytokines, cells were fixed and permeabilized with the FOXP3 Transcription Factor Staining Buffer Set according to the manufacturer’s protocol (Thermo Fisher Scientific). Dead cells were excluded using Fixable Viability Near-Infra Red Dye (1:1,000, Life Technologies, Thermo Fisher Scientific), Zombie Red Fixable Viability Kit (1:5,000, BioLegend), or a Zombie UV Fixable Viability Kit (1:500, BioLegend). Cytokine detection in tumor and LN single-cell preparations was performed following ex vivo stimulation in the presence of 1 μg/mL GolgiPlug (BD Biosciences) with 50 ng/mL PMA (MilliporeSigma) and 1 μM ionomycin (MilliporeSigma) dissolved in DMSO and diluted in 100 μL IMDM containing 8% FCS for 3 hours at 37°C, 5% CO_2_. Control (unstimulated) cells were treated with an equal volume of DMSO in the presence of GolgiPlug diluted in IMDM with 8% FCS. Absolute cell numbers were determined by adding AccuCount Blank Particles (7–7.9 μm, Spherotech) to each sample, prior to flow cytometric analysis. Fluorescence minus one (FMO) was used as a negative control for activation markers. Flow cytometry was performed using a BD FACSymphony A5 SORP flow cytometer or the 5-laser Cytek Aurora. All generated data were analyzed using FlowJo and OMIQ software (Dotmatics).

### Data analysis.

Dimensionality reduction and FlowSOM (88) analysis of flow cytometric data was performed using OMIQ software. Following conventional marker expression analysis, the cell population of interest was manually gated, and downsampling was performed to select the maximal number of cells per tissue representative for all tissue types included, as indicated in the figure legends. Tumor samples containing fewer than 600 cells of the subsampled population were excluded from analysis (see Figure 5D). K-means clustering of the indicated cell populations was performed using FlowSOM, including all markers indicated, except for live/dead and CD45 and for the CD8^+^ T cell population (see Figure 6, E and F) also without CD3. Dimension reduction and visualization were performed using UMAP analysis (89) and opt-SNE analysis (90), using the same markers as described above as well as the default OMIQ settings.

### Statistics.

All statistical data were analyzed using GraphPad Prism, version 9 (GraphPad Software). Ordinary 1-way ANOVA was performed when sample sizes were greater than 8, more than 3 experimental groups were compared, and if the assumption for normal distribution was met. If sample sizes were fewer than 8 and if normal distribution could not be assumed, Kruskal-Wallis analysis was applied. A *P* value of less than 0.05 was considered statistically significant. Error bars indicate the SD.

### Study approval.

Mice were maintained in individually ventilated cages (Innovive) under specific pathogen–free conditions. Only female mice were used to facilitate randomization of the large treatment groups. All mouse experiments were performed in accordance with institutional and national guidelines under license number AVD3010020173106 of the Central Committee for Animal Experiments (Centrale Commissie Dierproeven) and were approved by the Animal Welfare Body (IVD) of the Netherlands Cancer Institute.

### Data availability.

Data are available from the corresponding author upon request. Values for all data points found in graphs can be found in the Supplemental Supporting Data Values file.

## Author contributions

EF and J Borst conceived and designed the study. EF, TWB, and IV developed the study methodology. DMTB, TWB, J Busselaar, MDS, and IV provided advice on experiments. EF, DMTB, TWB, J Busselaar, and MDS acquired data. EF, TWB, and J Borst analyzed and interpreted data. EF and J Borst wrote the manuscript. DMTB, TWB, J Busselaar, MDS, and IV critically read and edited the manuscript.

## Supplementary Material

Supplemental data

Supplemental table 1

Supplemental table 2

Supporting data values

## Version 1 02/13/2024

In-Press Preview

## Version 2 03/15/2024

Electronic publication
