# Supplementary material for: PD-1 or CTLA-4 blockade promotes CD86-driven Treg responses upon radiotherapy of lymphocyte-depleted cancer in mice
Source: J Clin Invest. 2024 Feb 13;134(6):e171154. doi: 10.1172/JCI171154 (PMC10940086; doi:10.1172/JCI171154)
Supplement: Supplemental data [file jci-134-171154-s143.pdf]

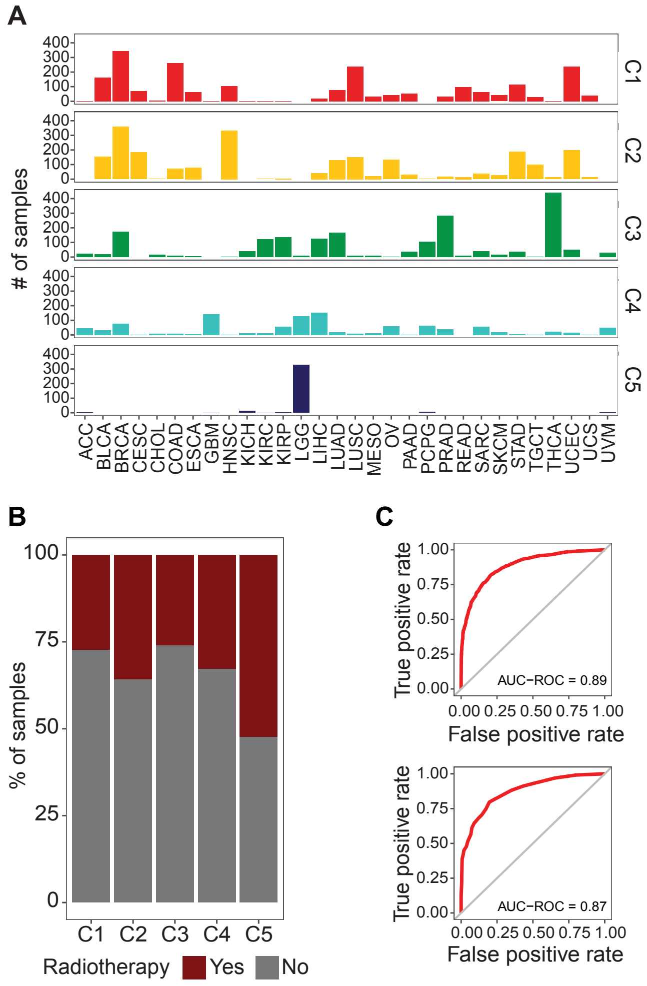


**Supplemental Figure 1- Related to Figure 1 (A – B).**

(**A**) Distribution of TCGA tumors (for tumor type abbrevation, see Supplemental Table 2) over the immune subtypes for which RT treatment (yes or no) was known. Bar heights indicate number of samples per tumor type. The total number of samples per tumor immune subtype (C1-C5) is indicated in Figure 1A. (**B**) Distribution of samples that received RT (red) or not (grey) for each tumor immune subtype. (**C**) Training (upper panel) and testing (lower panel) receiver operating curves (ROC) and area under the curve (AUC) calculation of a k-nearest neighbor's model training to classify C3 vs. C4/C5 TCGA immune subtypes. Training and testing were split 75% and 25%, respectively.

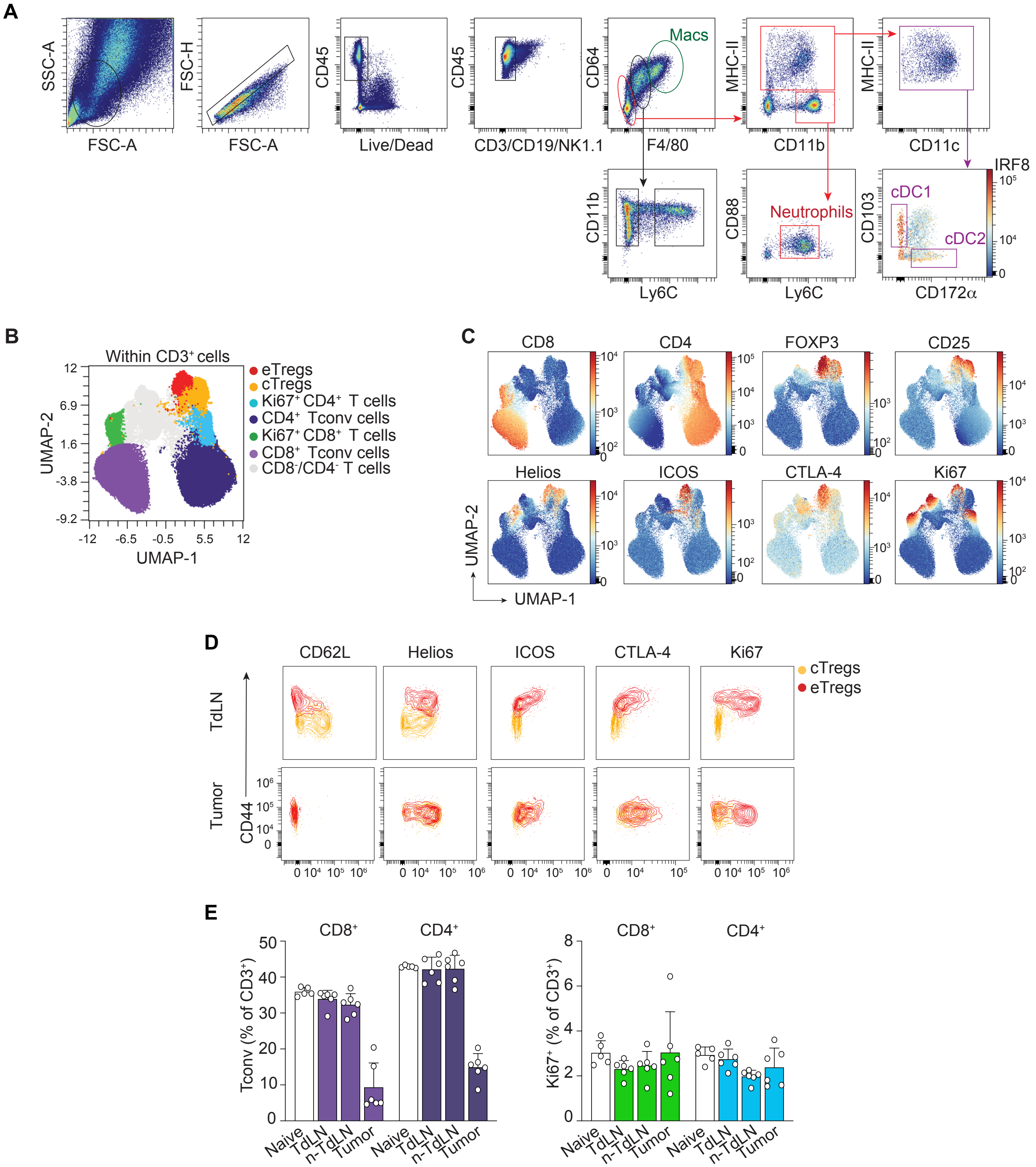


**Supplemental Figure 2 - Related to Figure 2 (A – E).**

(**A**) Representative gating strategy of the myeloid populations found in 50 mm$^2$ TC-1 tumors. (**B-C**) UMAP display of 5000 randomly selected CD3$^+$ cells per sample found in non-TdLN, TdLN and tumors at day 8 of all treatment groups combined. FlowSOM guided clustering (**B**) identifying the CD3$^+$ cell populations and (**C**) representative heat map visualization of the markers that identify the CD3$^+$ (T-cell) subpopulations. (**D**) Representative flow cytometry plots overlaying the in (**E**) identified cTreg and eTreg populations for TdLN (upper row) and tumor (lower row). (**E**) Percentage of the in (**B**) identified CD8$^+$ and CD4$^+$ Tconv populations (left) and Ki67$^+$CD8$^+$ and Ki67$^+$CD4$^+$ Tconv populations (right) among CD3$^+$ cells in the indicated tissues. Error bars indicate SD. Data is from one experiment, representative of two experiments.

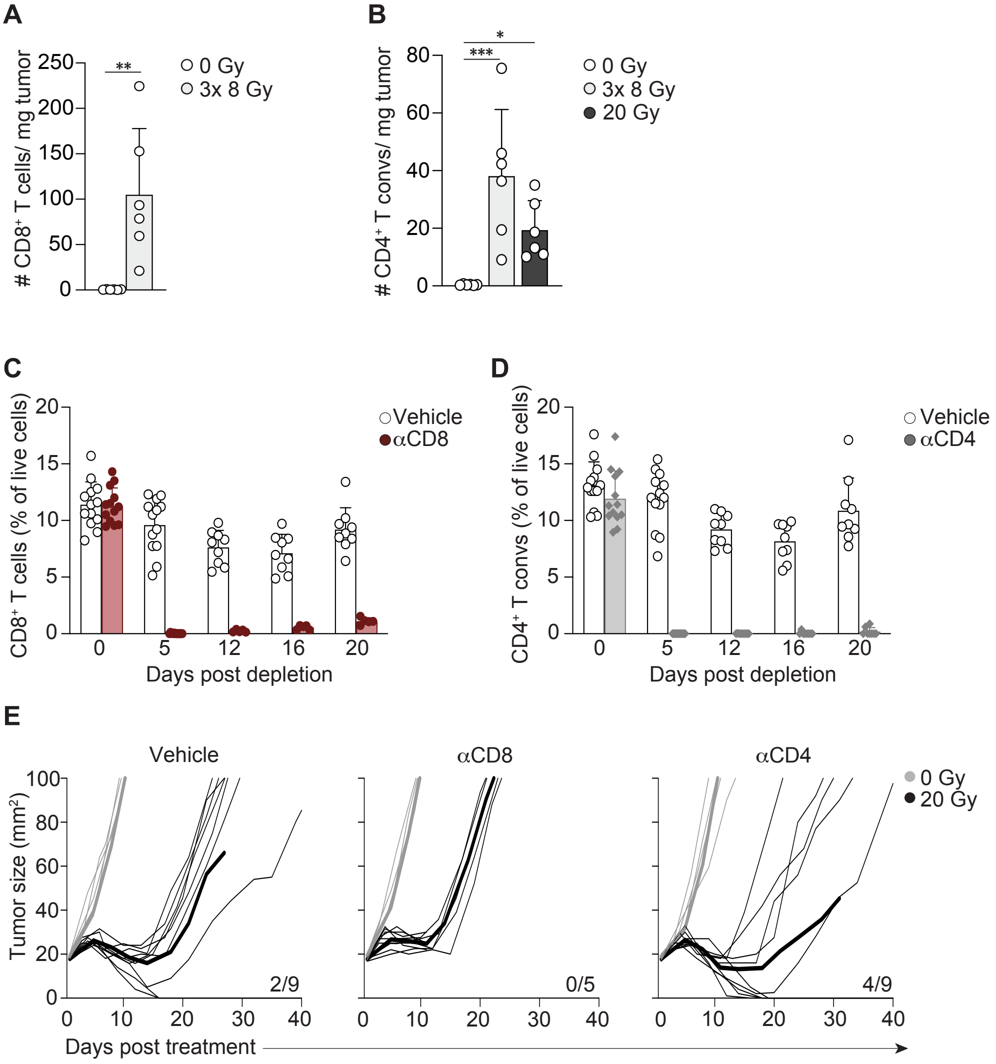


**Supplemental Figure 3 – Related to Figure 2 (F - H).**

(**A**) Absolute number (#) of total CD8+ T cells in TC-1 tumors treated with 8 Gy on three consecutive days (3x 8 Gy, n=6) or control (0 Gy, n=6) at day 8 post-RT. (**B**) Absolute number of (FOXP3-) CD4+ Tconv cells in TC-1 tumors treated with either 3x 8 Gy (n=6), 20 Gy (n=6) or control (0 Gy, n=6). (**C,D**) TC-1 tumor bearing mice were treated with 20 Gy RT (n=9/group) or control (0 Gy, n=4/group) at day 0 in combination with vehicle (PBS) or depleting mAbs against CD8 or CD4. (**C**) Frequency of CD8+ (left) or CD4+ Tconvs (right) among live cells in blood over time. The 0 Gy control group and 20 Gy groups are combined (Vehicle (PBS), n=13; αCD8, n=13; αCD4, n=13). (**D**) Individual tumor outgrowth curves belonging to Figure 2H. Thick lines indicate group averages. Data is from one experiment, representative of two experiments. Error bars indicate SD. *P < 0.05, **P < 0.01, *** P < 0.001, Mann-Whitney test in **A** and **B**. ns; not significant.

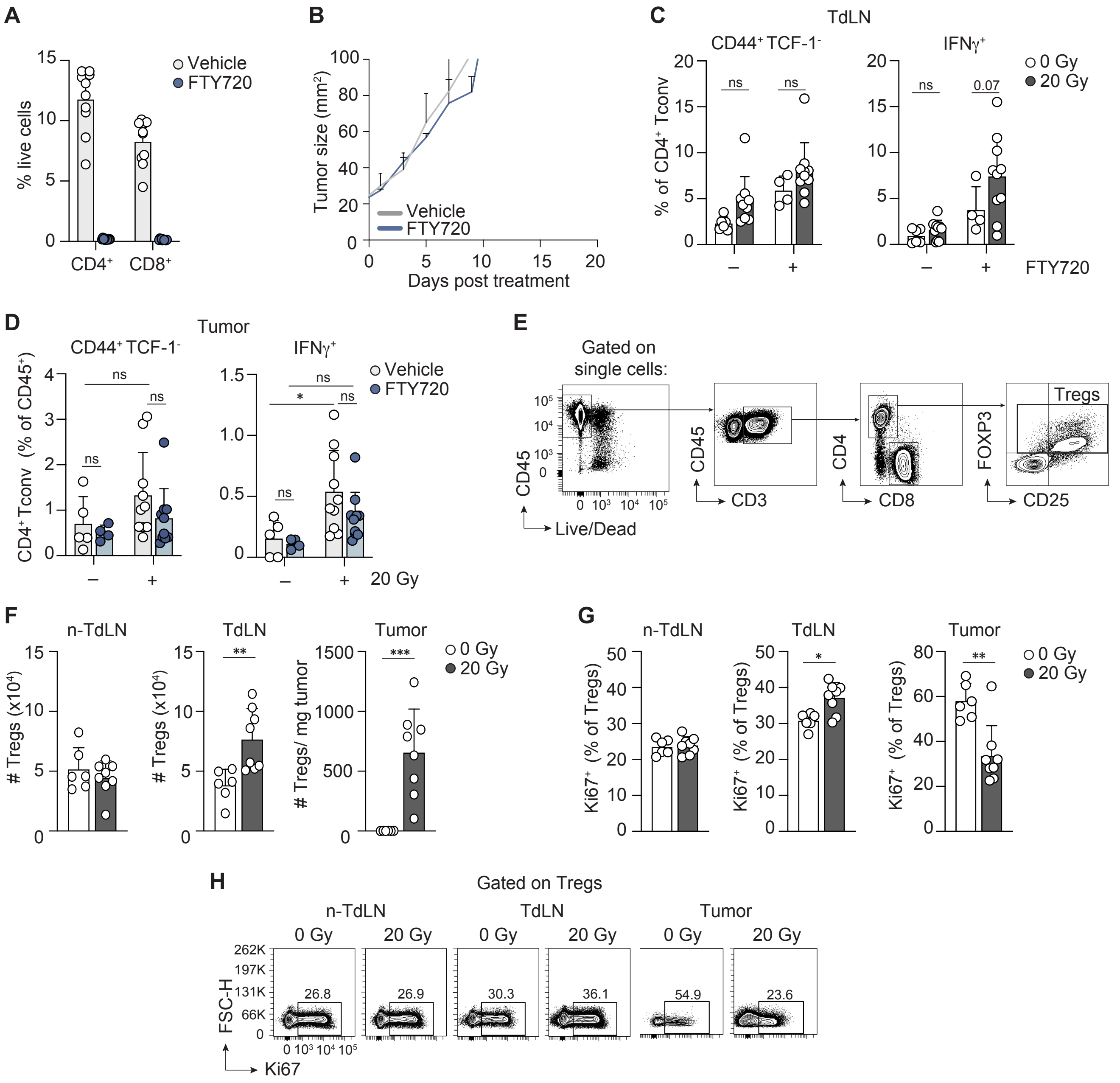


**Supplemental Figure 4 – Related to Figure 3 (A – H).**

(**A-D**) Data is from the same experiment described in Figure 3, A-D. TC-1 tumor bearing mice received 20 Gy RT (n=10/group) or 0 Gy (n=4-6/group) when tumor sizes reached ~20 $mm^2$ (day 0) in combination with FTY720 or vehicle (NaCl) by oral gavage, starting at day -1 and followed by days 3 and 5. At day 8, the TdLN and tumor were isolated and the ($FOXP3^-$) $CD4^+$ T cell response was analyzed. (**A,B**) Frequency of $CD4^+$ and $CD8^+$ Tconvs among live cells in blood at day 7 (**A**) and average tumor outgrowth curves (**B**) in TC-1 tumor bearing mice treated with 0 Gy and FTY720 or vehicle. (**C,D**) Frequency of $CD44^+$ TCF-$1^-$ and IFN$\gamma^+$ cells among $CD4^+$ T cells in the TdLN (**C**) and in the tumor (**D**) of mice treated with 0 Gy or 20 Gy.(**E-G**) Monitoring of the ($FOXP3^+$ $CD25^+$) Treg response to 20 Gy RT (n=8) or 0 Gy (n=6) in TC-1 tumor bearing mice by flow cytometry at day 8 post treatment. (**E**) Representative gating strategy of Treg cells, based on $FOXP3^+$ and $CD25^+$ expression. (**F**) Absolute counts (#) of total Treg cells in the non-TdLN, TdLN and tumor. (**G**) Percentage of $Ki67^+$ cells among Tregs in the indicated tissues. (**H**) Representative concatenated (0 Gy, n=6; 20 Gy, n=8) flow cytometry plots depicting $Ki67^+$ cells among Treg cells found in the non-TdLN, TdLN and tumor at day 8 post treatment. Numbers indicate the percentage of $Ki67^+$ cells. Data is from one experiment, representative of two experiments. Error bars indicate SD. $^*P < 0.05$, $^{**}P < 0.01$, $^{***}P < 0.001$, two-way Anova with Tukey's post hoc test in **C** and **D**, Mann-Whitney test in **F** and **G**. ns; not significant.

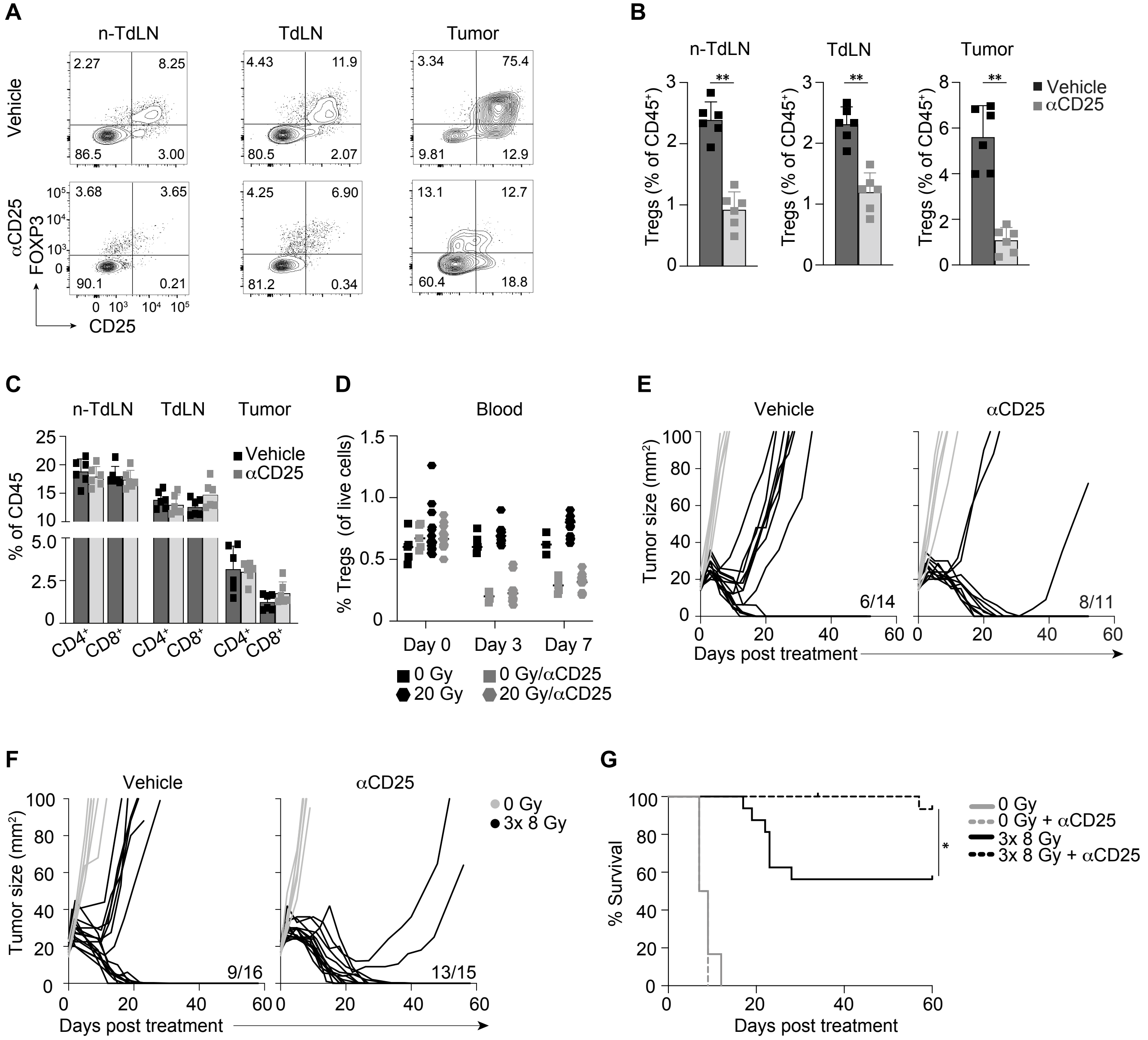


**Supplemental Figure 5 - Related to Figure 3(I).**
(**A-C**) TC-1 tumor bearing mice were treated i.p. with a depleting mAb against CD25 (n=6) or vehicle (PBS, n=6) when tumor size reached ~20 mm$^2$ (day 0). At day 4, the non-TdLN, TdLN and tumor were harvested and the Treg response was analyzed by flow cytometry. (**A**) Representative flow cytometry plots and (**B**) quantification depicting the frequency of total Tregs in the indicated tissues. (**C**) Proportion of CD4$^+$ and CD8$^+$ Tconvs among CD45$^+$ cells found in the indicated tissues following treatment. (**D**) Frequency of total Tregs among live cells measured in blood over time in the indicated treatment groups and (**E**) the individual tumor growth curves of the data described in Figure 3I**.** (**F**) **I**ndividual tumor growth curves and (**G**) overall survival of TC-1 tumor bearing mice treated with 0 Gy (n=6) or 3x8 Gy (n =15-16) in combination with a depleting mAb against CD25 or vehicle (PBS) at day -1 and 5 post RT. Ratios indicate the number of mice that showed full recovery upon treatment. Data are from one experiment representative of two experiments. Error bars indicate SD. *P < 0.05, **P < 0.01, Mann-Whitney test in **B**, Mantel-Cox analysis in **G** and Kruskal-Wallis test with Dunn's post hoc test in **H.** ns; not significant.

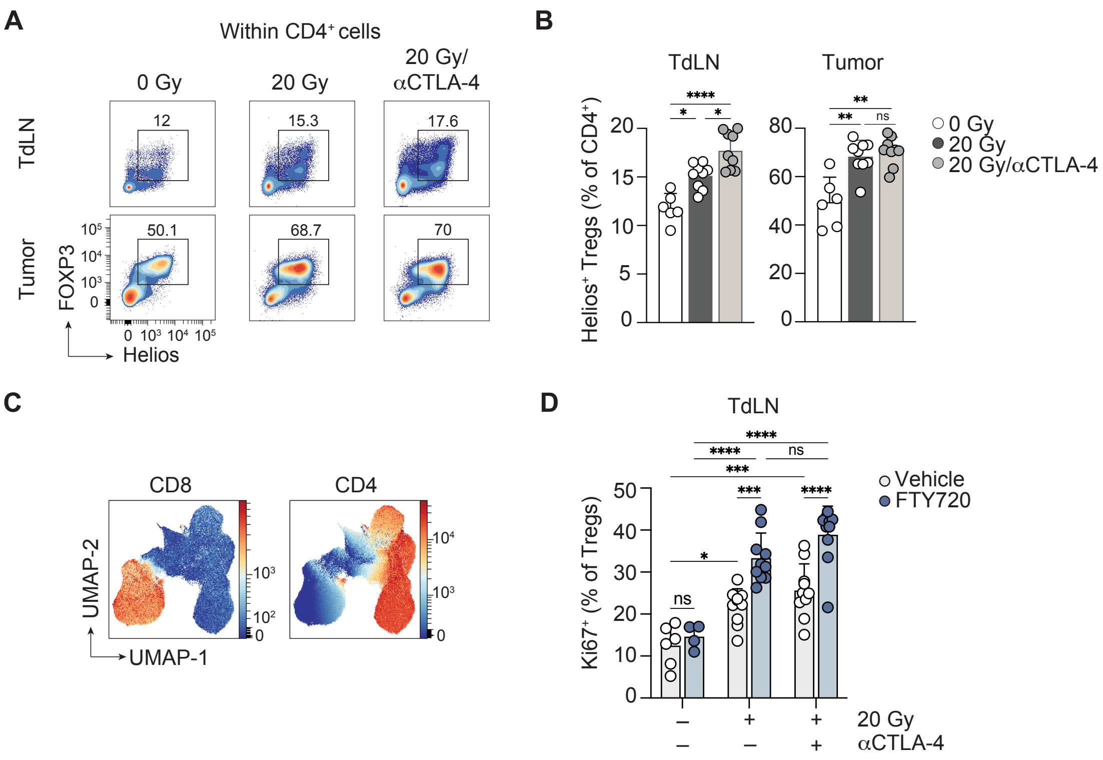


**Supplemental Figure 6 – Related to Figure 4.**
(**A-C**) Mice bearing 20 mm$^2$ TC-1 tumors received RT (20 Gy, n=9) or control (0 Gy, n=6) on day 0. Treatment included vehicle (PBS) or a CTLA-4 blocking mAb on day 0, 3 and 6. The Treg response was measured by flow cytometry in the indicated tissues on day 8. (**A**) Representative concatenated flow cytometry plots and (**B**) quantification depicting the frequency of Helios$^+$ Tregs among CD4$^+$ T cells in the TdLN and tumor. (**C**) UMAP visualization of the CD8$^+$ cells and CD4$^+$ cells among CD3$^+$ cells, related to Figure 4, D and E. (**D**) TC-1 tumor-bearing mice received 20 Gy (n=10/group) or control (0 Gy, n=4-6), with CTLA-4 mAb blockade or vehicle on days 0, 3, and 6, with or without FTY720. Depicted is the frequency of Ki67$^+$ cells among Tregs measured in the TdLN on day 8 post-RT.
Data are from one experiment representative of two experiments. Error bars indicate SD. *P < 0.05, **P < 0.01, *** P < 0.001, **** P < 0.0001, Kruskal-Wallis with Dunn's post hoc test in **B**, two-way Anova with Tukey's post hoc test in **D**. ns; not significant.

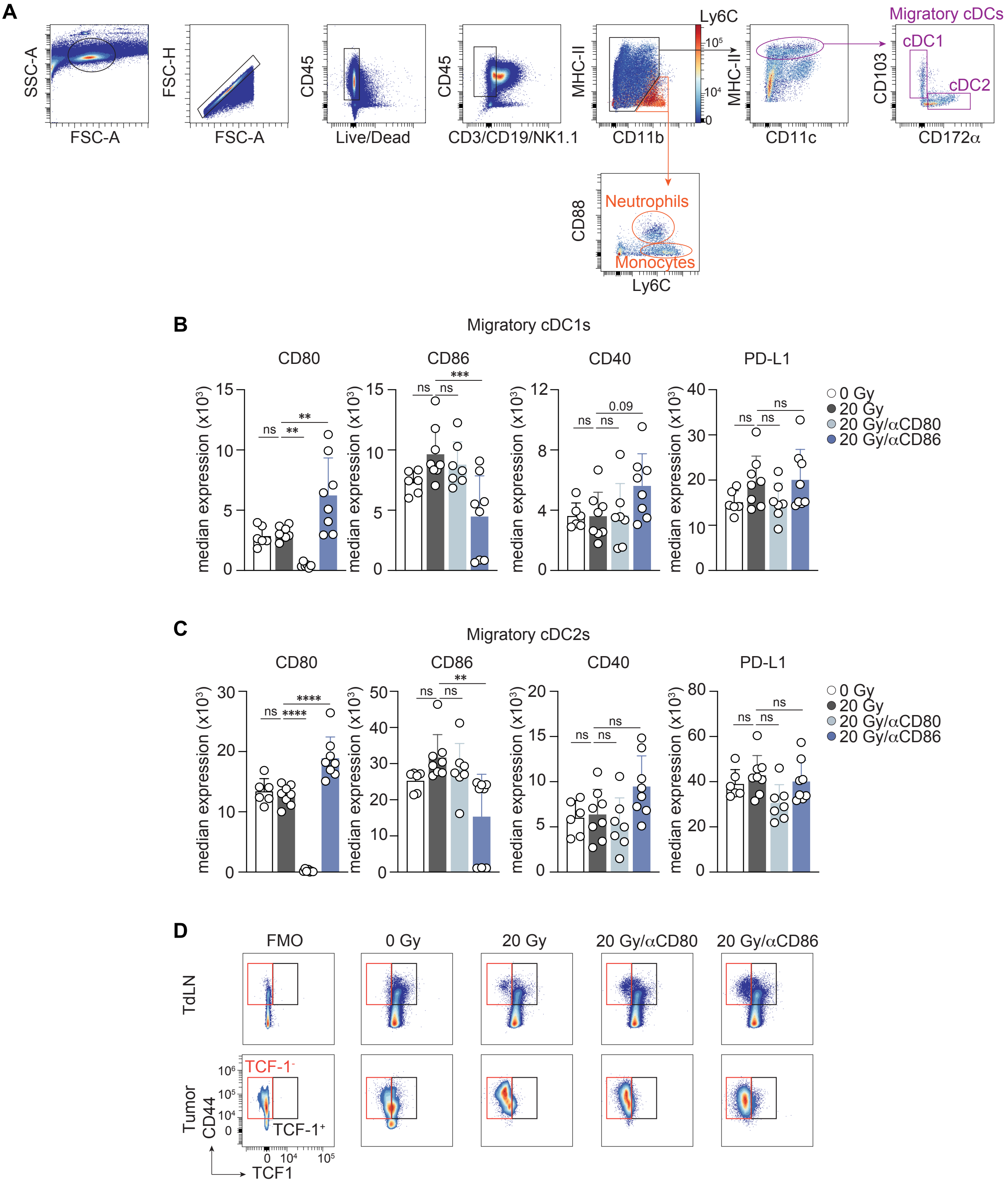


**Supplemental Figure 7 – Related to Figure 6.**

(**A**) Representative gating strategy of cDC subsets in the TdLN of TC-1 tumor bearing mice. (**B**,**C**) Mice bearing 20 $mm^2$ TC-1 tumors received control treatment (0 Gy, n=6) or 20 Gy RT at day 0 in combination with either vehicle (PBS, n=8) or blocking mAb against CD80 (n=7) or CD86 (n=8) at day 0, 3 and 6. The cDC response was monitored by flow cytometry in the TdLN at day 8. (**B**,**C**) Median expression of the indicated markers found on (**B**) migratory cDC1s and (**C**) migratory cDC2s in the TdLN. (**D**) Representative gating strategy of the $CD44^+$ $TCF\text{-}1^-$ cells (orange) and $CD44^+$ $TCF\text{-}1^+$ cells (black) among $CD8^+$ T cells in the TdLN (upper row) and tumor (lower row) for the indicated treatment groups at day 8. FMO; fluorescence minus one. Data are from one experiment representative of two experiments. Error bars indicate SD. **P < 0.01, *** P < 0.001, **** P < 0.0001, ordinary one-way Anova with Dunnett's post hoc test in **C, D**. ns; not significant.

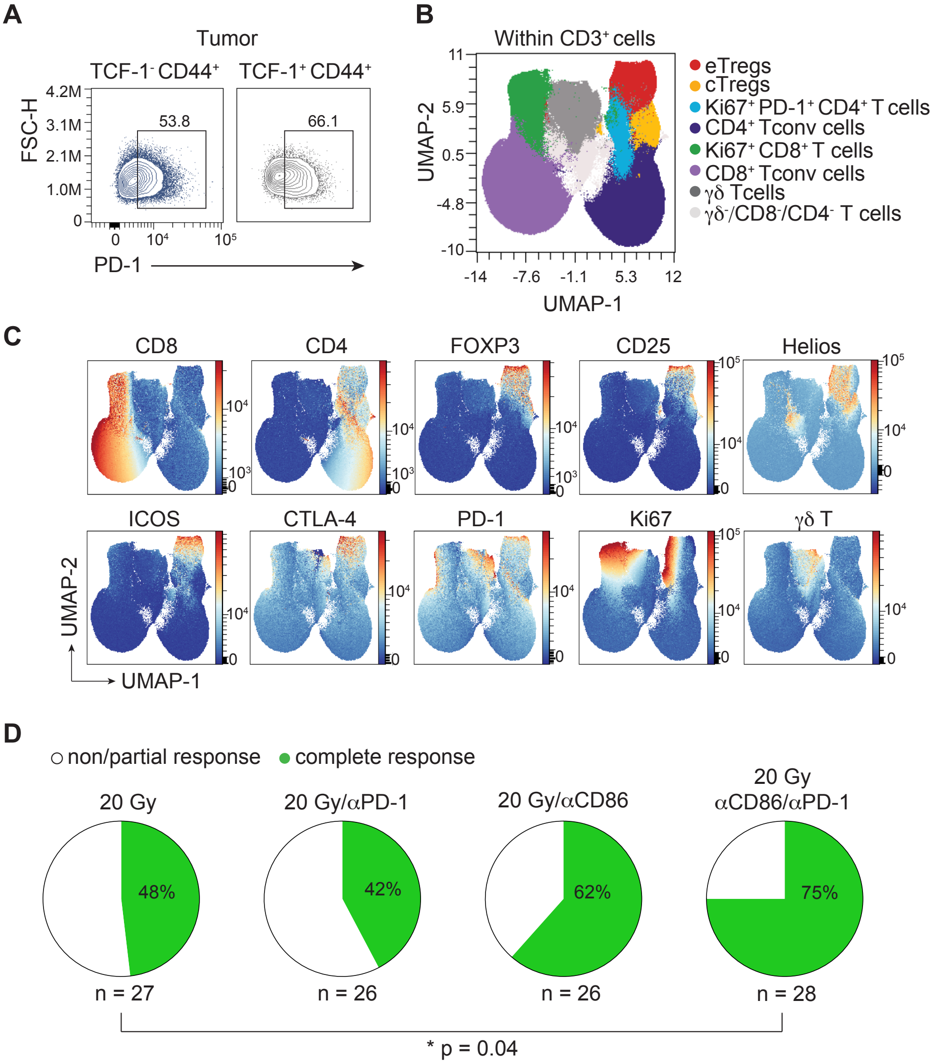


**Supplemental Figure 8 – Related to Figure 7.**

(**A**) Mice bearing 20 mm$^2$ TC-1 tumors received control treatment (0 Gy, n=5) or 20 Gy RT at day 0 in combination with either vehicle (PBS, n=8) or blocking mAb against CD80 (n=11) or CD86 (n=11) at day 0, 3 and 6. The CD8$^+$ T cell response was monitored by flow cytometry in the tumor at day 8. Representative concatenated (n=11) contour plots are depicted for PD-1 expression on the indicated cell populations among CD8$^+$ T cells in mice treated with 20 Gy and CD86 blockade. Numbers indicate percentages. (**B-C**) Mice bearing TC-1 tumors received control treatment (0 Gy, n=4) or 20 Gy RT at day 0 in combination with either vehicle (PBS, n=8) or blocking mAb against PD-1 (n=11), CD86 (n=10) or a combination of both (n=10) at day 0, 3 and 6. The CD3$^+$ (T-cell) lymphocyte response was monitored by flow cytometry in the non-TdLN, TdLN and tumor at day 8. UMAP display of 2500 randomly selected CD3$^+$ cells per tissue found in non-TdLN, TdLN and tumors at day 8 of all treatment groups combined. FlowSOM guided clustering (**B**) identifying the CD3$^+$ cell populations and (**C**) representative heat map visualization of the markers that identify the CD3$^+$ subpopulations. (**D**) Pie chart depicting the proportion of TC-1 tumor-bearing mice with complete tumor clearance upon treatment with 20 Gy at day 0, in combination with either vehicle (PBS), or blocking mAb against PD-1, CD86 or both CD86 and PD-1 at day 0, 3 and 6. *P<0.05, Chi-square test.
